# Synthesis and Antiproliferative Activity of Minor Hops Prenylflavonoids and New Insights on Prenyl Group Cyclization

**DOI:** 10.3390/molecules23040776

**Published:** 2018-03-28

**Authors:** Jarosław Popłoński, Eliza Turlej, Sandra Sordon, Tomasz Tronina, Agnieszka Bartmańska, Joanna Wietrzyk, Ewa Huszcza

**Affiliations:** 1 Department of Chemistry, Wrocław University of Environmental and Life Sciences, Norwida 25, 50-375 Wrocław, Poland; Sandra.sordon@upwr.edu.pl (S.S.); Tomasz.tronina@upwr.edu.pl (T.T.); Agnieszka.bartmanska@upwr.edu.pl (A.B.); Ewa.Huszcza@upwr.edu.pl (E.H.); 2 Department of Experimental Oncology, Hirszfeld Institute of Immunology and Experimental Therapy, Polish Academy of Sciences, Weigla 12, 53-114 Wrocław, Poland; elizaturlej@interia.pl (E.T.); wietrzyk@iitd.pan.wroc.pl (J.W.)

**Keywords:** xanthohumol, isoxanthohumol, chalcone, cyclisation, antiproliferative activity, hops, spent hops

## Abstract

Synthesis of minor prenylflavonoids found in hops and their non-natural derivatives were performed. The antiproliferative activity of the obtained compounds against some human cancer cell lines was investigated. Using xanthohumol isolated from spent hops as a lead compound, a series of minor hop prenylflavonoids and synthetic derivatives were obtained by isomerization, cyclisation, oxidative-cyclisation, oxidation, reduction and demethylation reactions. Three human cancer cell lines—breast (MCF-7), prostate (PC-3) and colon (HT-29)—were used in antiproliferative assays, with cisplatin as a control compound. Five minor hop prenyl flavonoids and nine non-natural derivatives of xanthohumol have been synthetized. Syntheses of xanthohumol K, its dihydro- and tetrahydro-derivatives and 1″,2″,α,β-tetrahydroxanthohumol C were described for the first time. All of the minor hops prenyl flavonoids exhibited strong to moderate antiproliferative activity in vitro. The minor hops flavonoids xanthohumol C and 1″,2″-dihydroxanthohumol K and non-natural 2,3-dehydroisoxanthohumol exhibited the activity comparable to cisplatin. Results described in the article suggest that flavonoids containing chromane- and chromene-like moieties, especially chalcones, are potent antiproliferative agents. The developed new efficient, regioselective cyclisation reaction of the xanthohumol prenyl group to 1″,2″-dihydroxantohumol K may be used in the synthesis of other compounds with the chromane moiety.

## 1. Introduction

Flavonoids are a group of secondary plant metabolites and important food constituents with a daily intake of a few hundreds of milligrams and positive effects on human health [1]. Their anticancer activity observed at the initiation, promotion, and progression stages of cancer is considered to be a most promising biological phenomenon [2,3]. Currently, there is high interest in direct use of flavonoids as anticancer drugs [3,4] or as enhancers for existing anticancer therapies [5,6].

Hops, the female inflorescences of *Humulus lupulus*, are a natural source of numerous compounds that exhibit a remarkably broad spectrum of biological activities [7,8,9]. One of the best known compounds isolated from hops is 3′-[3,3-dimethylallyl]-2′,4′,4-trihydroxy-6′-methoxy- chalcone (xanthohumol, **1**), which exhibits various biological properties such as anticancer, anti-inflammatory, central nervous systems modulation, antimicrobial, anti-parasite, anti-obesity, hepatic protection and antioxidant effects [7,8,9,10]. Apart from xanthohumol, many other xanthohumol derivatives, abbreviated with capital letters from B to M, have been identified in hops so far [8,11,12,13]. Little is known however about their biological activities [13,14,15,16,17], mainly due to the fact that only trace amounts of these compounds are found in the plant material. Xanthohumol′s extraordinary biological properties have given rise to a couple of reports concerning chemical [13,14,15,16,17,18,19,20] and microbial [21,22,23] modifications of these compounds that might increase its potency. Few of them, however, relate to the synthesis and biological activity [15,17].

While there is a great interest in developing new synthetic flavonoid-like compounds, the biological potential of many of the known flavonoids remains unevaluated. This is especially important with respect to the fact that these compounds can be, or already are, a part of our diet. Recently, a few reports have stated that chalcones containing a dimethylchromene moiety can be more effective anti-inflammatory agents than the common anti-inflammatory drug indometacin [24] or exhibit lower cytotoxicity towards normal cell lines than non-cyclized derivatives [20]. Furthermore, some modified chalcones with cyclized prenyl groups exhibit strong antiproliferative activity in vitro and in vivo [25,26].

Our previous report concerning the antiproliferative activity of xanthohumol microbial metabolites revealed that α,β-dihydroxanthohumol, which is found in trace amounts in hops, was more active than xanthohumol (**1**) [23]. This finding led us to investigate biological activity of other xanthohumol compounds, along with an assessment of their structure-activity relationships. Herein we wish to report the synthesis and biological evaluation of a series of xanthohumol-derived compounds with cyclized prenyl groups. Moreover, we would like to present some interesting synthetic procedures allowing direct and regioselective cyclisation of prenyl groups that have not yet been reported.

## 2. Results and Discussion

Previous reports on the synthesis of minor hops prenylchalconoids by Vogel and Heilmann [15] suggested that there were only slight differences among xanthohumol derivatives when comparing their cytotoxic activity against HeLa cells. However, cyclized xanthohumol C (**2**) has better activity than its 1″,2″-dihydroderivative **3** [15]. Because the purpose of our research was to obtain derivatives of xanthohumol (**1**) with a cyclic prenyl group, we isolated our primary compound **1** from spent hops, which is a rich source and it can be easily purified in considerable amounts [27].

**Scheme 1:**
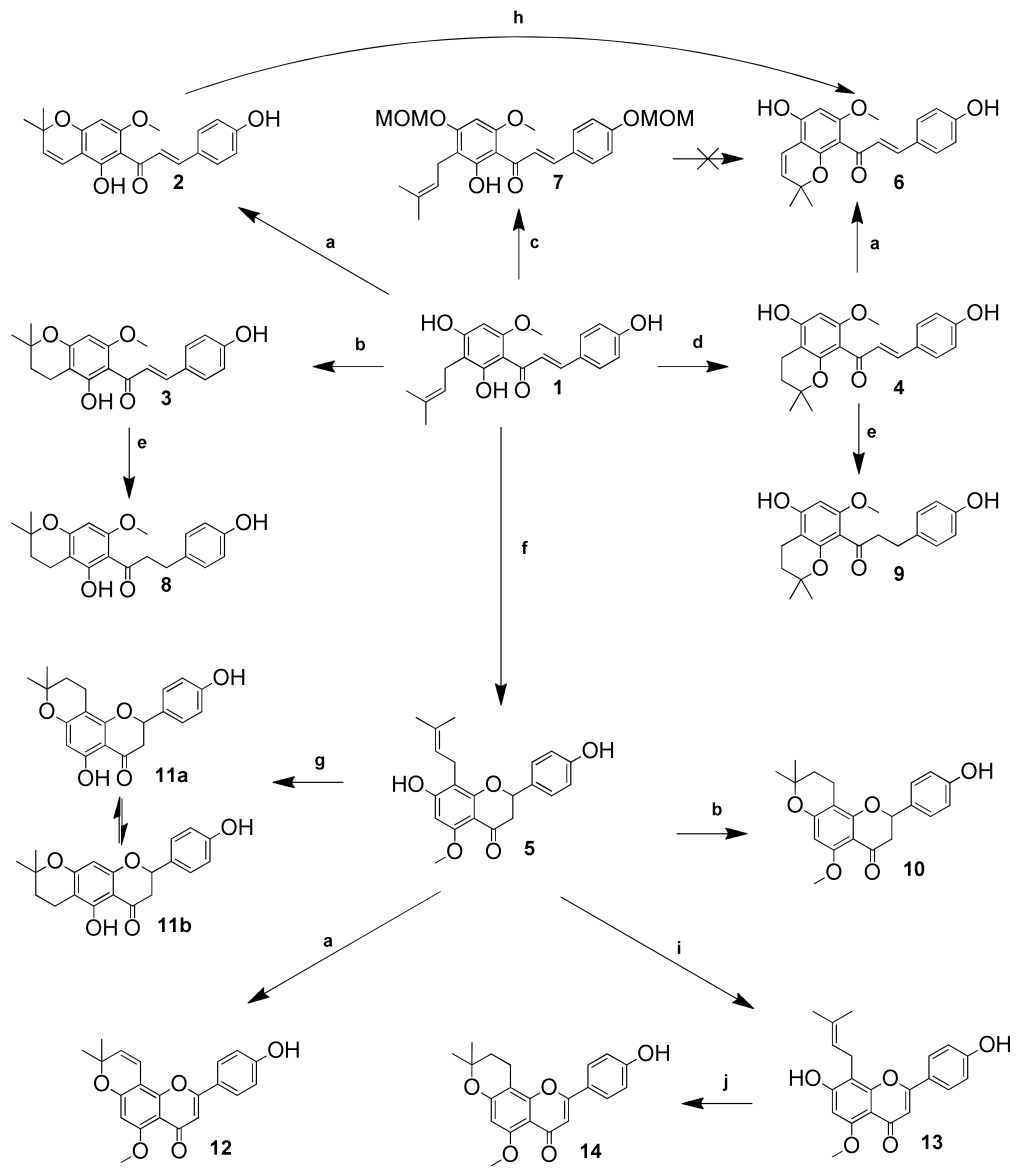
General synthesis scheme of xanthohumol derived compounds. Reagents and conditions: (a) DDQ, 1,4 dioxane, 95 °C (b) AlCl_3_, CH_2_Cl_2_, rt; (c) MOMCl, K_2_CO_3_, acetone, rt; (d) TFA, CH_2_Cl_2_, rt; (e) H_2_, 5% Pd/C, MeOH, rt; (f) *aq* 1% KOH, 0 °C; (g) AlCl_3_, MeCN, 50 °C; (h) *aq* 1% piperidine reflux (i) I_2_, pyridine, 90 °C; (j) anhydrous AlCl_3_, MeCN, rt.

Modifying the procedure described by Vogel and Heilmann [15] for the reaction of **1** and 2,3-dichloro-5,6-dicyano-1,4-benzoquinone (DDQ) in anhydrous 1,4-dioxane at 95 °C we obtained **2** in 74% yield (Scheme 1). By application of straightforward chemistry, without protection of hydroxyl groups, we were expecting to observe traces of other cyclization products, since in **1** there are two adjacent hydroxyl groups that could undergo cyclization to a dimethylchromene moiety. However, no other cyclisation products were detected in the reaction mixture, which is in agreement with other reports concerning DDQ cyclisation of prenyl groups and reaffirms the selectivity of the reaction [28].

1″,2″-Dihydroxanthohumol C (**3**) was obtained using the modified method described by Nerander and Papi Reddy [29]. Xanthohumol (**1**) was stirred with anhydrous aluminum chloride in methylene chloride (DCM) for 5 h at room temperature to afford **3** in a yield of 87% (Scheme 1). It is noteworthy that in this reaction no side products (for example **4**) were formed. The modification associated with the use of an anhydrous AlCl_3_, instead of the boron trifluoride diethyl etherate, was due to strong complexation of boron trifluoride, which caused problems for its removal, and reduced the final yield of **4** (42–53% yield). In turn reactions with other Lewis acids, like ZnCl_2_, SnCl_2_, SnCl_4_, PdCl_2_ also were characterized by low yields of **3** and did not lead to formation of product **4**. This phenomenon can be explained by a possible complexation of the hydroxyl group at C-2′ and the carbonyl oxygen atom preventing other side products from being formed, not only prenyl group cyclisation, but also the flavanone compound—isoxanthohumol (**5**).

Since our investigation focused on other new prenyl group cyclisation products **4** or **6** and at this point we did not find literature data concerning methods allowing to change the regioselectivity of cyclisation, therefore we performed series of reactions in which reagent amounts (0.3–3.0 equiv.), temperatures (0 °C, 20 °C, 50 °C) and solvents (THF, DCM, DMF, DMSO) were modified. These efforts also failed to provide any detectable amounts of the desired compounds. In the next approach we tried to selectively block the phenolic groups at C-4 and C-4′, leaving only one possible site of cyclisation. The protection was achieved using methoxymethyl chloride (MOMCl) with K_2_CO_3_ in acetone to give **7** (Scheme 1) by a slow and controlled addition of MOMCl monitored by HPLC. It is worth noting that even with an elevated molar ratio of MOMCl the only product formed by this method was **7**. Another method involving *N*,*N*-diisopropylethylamine led to mixtures, and finally the fully protected compound.

Interestingly, the subsequent oxidative cyclisation of **7** with DDQ failed to give the expected protected derivative of **6**, even when increased amounts of DDQ were applied. However, deprotection with trifluoroacetic acid (TFA) in DCM led to another interesting product. Purification and spectroscopic analysis proved it was **4**, instead of **6**. This encouraged us to perform a series of reactions to test this catalyst with **1**. The reaction of **1** with TFA (1% *v*/*v*) in DCM at room temperature afforded mainly **4** (71%), along with a small amount of **3** (18%) (Scheme 1). Noteworthy, so far there are no reports concerning direct cyclisation of the prenyl moiety to the C-2′ hydroxyl group in the presence of a free hydroxyl group at C-4′, the synthetic method leading from **1** to **4**. Therefore, we carried out a few experiments to test the preliminary conditions and to find an explanation for such a phenomenon (Table 1).

**Scheme 2:**
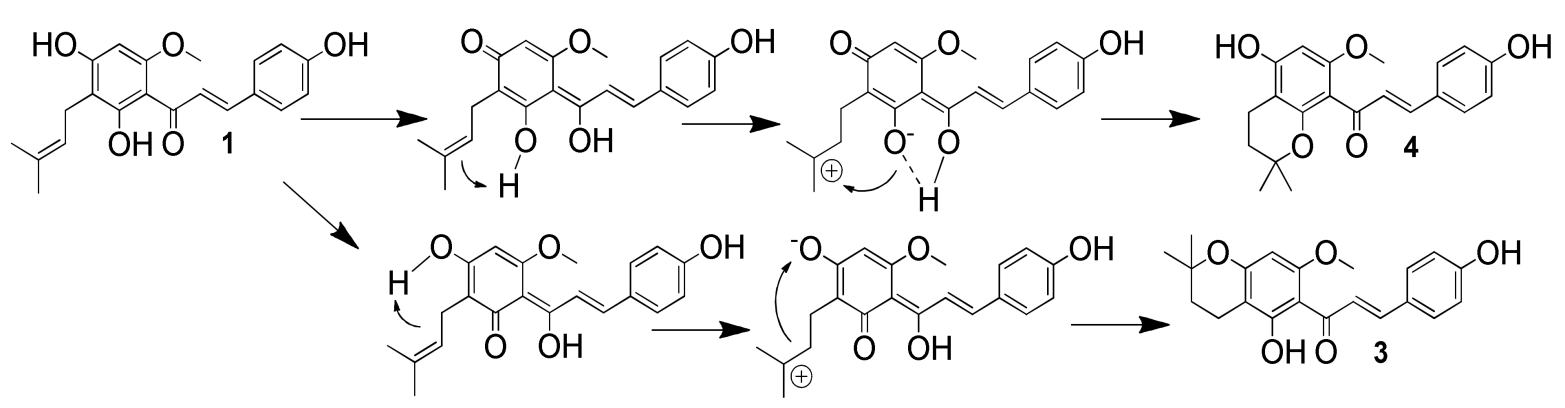
Proposed mechanism of cyclisation of xanthohumol (**1**) to 1″,2″-dihydroxanthohumol K (**4**) and 1″,2″-dihydroxanthohumol C (**3**) by TFA.

The results presented in Table 1 show that an increased temperature and a decreased amount of TFA favor cyclisation to **4** over **3**. Obviously, increasing the temperature and TFA amount improved the reaction yield, as well. The proposed mechanism is shown in Scheme 2. This mechanism proposal is also supported by the fact, that by using the same procedure with other organic acids (formic acid, acetic acid, propionic acid, oxalic acid, maleic acid and picric acid) added in 1% (*v*/*v*) or molar equivalent to 1% of TFA did not lead to compound **4**, what was also observed by Andrade-Carrera et al. [28]. Only in case of trichloroacetic acid and prolonged reaction times were traces of **4** found. Reactions with inorganic acids (H_2_SO_4_, H_3_PO_4_) led to isomerisation of **1** to **5** with formation of many other side products. Presumably, under anhydrous conditions TFA acts as an acid-base catalyst allowing intramolecular hydrogen transfer (Scheme 2). Thus, the first step involves protonation of the carbonyl group by TFA and further deprotonation of one of hydroxyl groups in ring A leading to the formation of a quinone-like intermediate. Then, protonation of the double bond with a hydrogen from a hydroxyl group (C-2′ or C-4′), formation of the tertiary carbocation and cyclisation with a nucleophilic oxygen followed by a quinone to phenol rearrangement supported by TFA via protonation and deprotonation. Possible hydrogen bond formed with an adjacent enol might stabilize the oxygen anion at C-2′, what might hinder further protonation by TFA and may be responsible for the regioselectivity of the proposed mechanism. Nevertheless, more efforts have to be made to clarify and understand this unusual cyclisation. It should be noted that the only report found describing similar prenyl group cyclisation are studies of 6-prenylnaringenin by Mizobuchi who applied TFA in chloroform as a method of unequivocal differentiation between 6- and 8-prenylnaringenin, but without any comments or further investigation however [30].

Since compound **4** can be easily prepared, we decided to obtain **6** by dehydrogenation of the dimethylchromane ring. Thus we employed several dimethylchromane ring dehydrogenation methods using SeO_2_ and DDQ, but only the reaction with excess of DDQ in benzene or 1,4-dioxane resulted in formation of **6**. A preparative scale dehydrogenation reaction was conducted using the same method as for the synthesis of **2** (Scheme 1) and it provided **6** in a yield of 17%.

Given the results of our previous studies indicating that antiproliferative activity of the xanthohumol microbial metabolite α,β-dihydroxanthohumol was comparable to that of xanthohumol, [23] we decided to evaluate the impact of the α,β-unsaturated bond on the antiproliferative activity of α,β-dihydrochalcone derivatives of **3** and **4**. Thus, **3** and **4** were hydrogenated using a previously described method [31] with 5% palladium on charcoal in methanol to afford 1″,2″,α,β-tetrahydroxanthohumol C (**8**) and 1″,2″,α,β-tetrahydroxanthohumol K (**9**) in good yields (91% and 88%, respectively, Scheme 1).

**Scheme 3:**
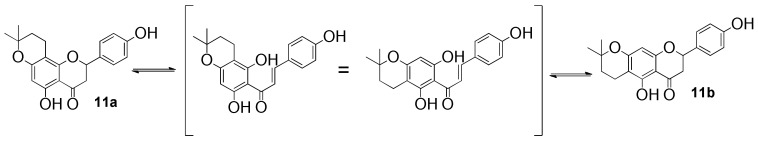
Chalcone-flavanone isomerization of 11a to 11b.

In order to study the effect of cyclization on the antiproliferative activity of different classes of flavonoids (flavanones and flavones) we also obtained isoxanthohumol (**5**) by the base catalysed isomerisation of xanthohumol (**1**) using the procedure previously reported by Bartmańska et al. [27] (Scheme 1). Subsequently application of reaction conditions similar to those for the synthesis of **3** afforded 1″,2″-dihydroisoxanthohumol C (**10**) in 76% yield from isoxanthohumol (**5**) as a substrate (Scheme 1). Modification of the reaction conditions involving the use of acetonitrile, increased temperature and extended reaction time resulted in cyclization followed by demethylation of **10** to give **11a** in 80% yield. Interestingly, TLC and HPLC analysis of the reaction mixture indicated the formation of only one product, but further NMR analysis clearly indicated that the product obtained after chromatographic purification on silica gel is a mixture of two compounds **11a** and **11b** (Scheme 3). The analytical methods applied did not unambiguously determine the **11a** and **11b** ratio in the mixture. Nevertheless, such determination would probably not be useful, since literature data presented by Simmler et al. [32] indicates that chalcone-flavanone isomerization leading to isomerization between **11a** and **11b** is inevitable during biological assays like in vitro cytotoxicity tests, therefore we decided to use the mixture of compounds **11a** and **11b** in the subsequent antiproliferative activity assay.

The next step on our synthetic pathway was to obtain a product with a dimethylchromene moiety from isoxanthohumol (**5**), thus cyclization with DDQ was the method of choice. In order to obtain isoxanthohumol C, a number of reactions were performed with different DDQ amounts (0.3–1.2 equiv.) temperatures (0–100 °C) and addition of DDQ over time, but in all cases cyclization of the prenyl group occurred simultaneously with dehydrogenation of C2-C3 bond resulting in 2,3-dehydroisoxanthohumol C (**12**). A reaction set up for the complete conversion of **5** resulted in **12** in 76% yield (Scheme 1). Another synthetic approach was to isomerize xanthohumol C (**2**) to isoxanthohumol C. Reactions with sodium and potassium hydroxides or with sodium acetate in ethanol [33] did not lead to the formation of desired product. Even prolonged reaction times (over 2 days), resulted in mixtures of many side products, therefore, we applied organic bases as catalysts, as such procedures in some cases seems to be more effective [34,35]. Reactions in water with addition of 2% (*v*/*v*) diethylamine, diisopropylamine, triethylamine, pyridine, pyrrolidine and piperidine carried out at room temperature for up to 48 h did not result in any flavanone compounds. In the reaction with piperidine small amounts of the unexpected xanthohumol K (**6**) were found (18% conversion, Scheme 1).

**Scheme 4:**
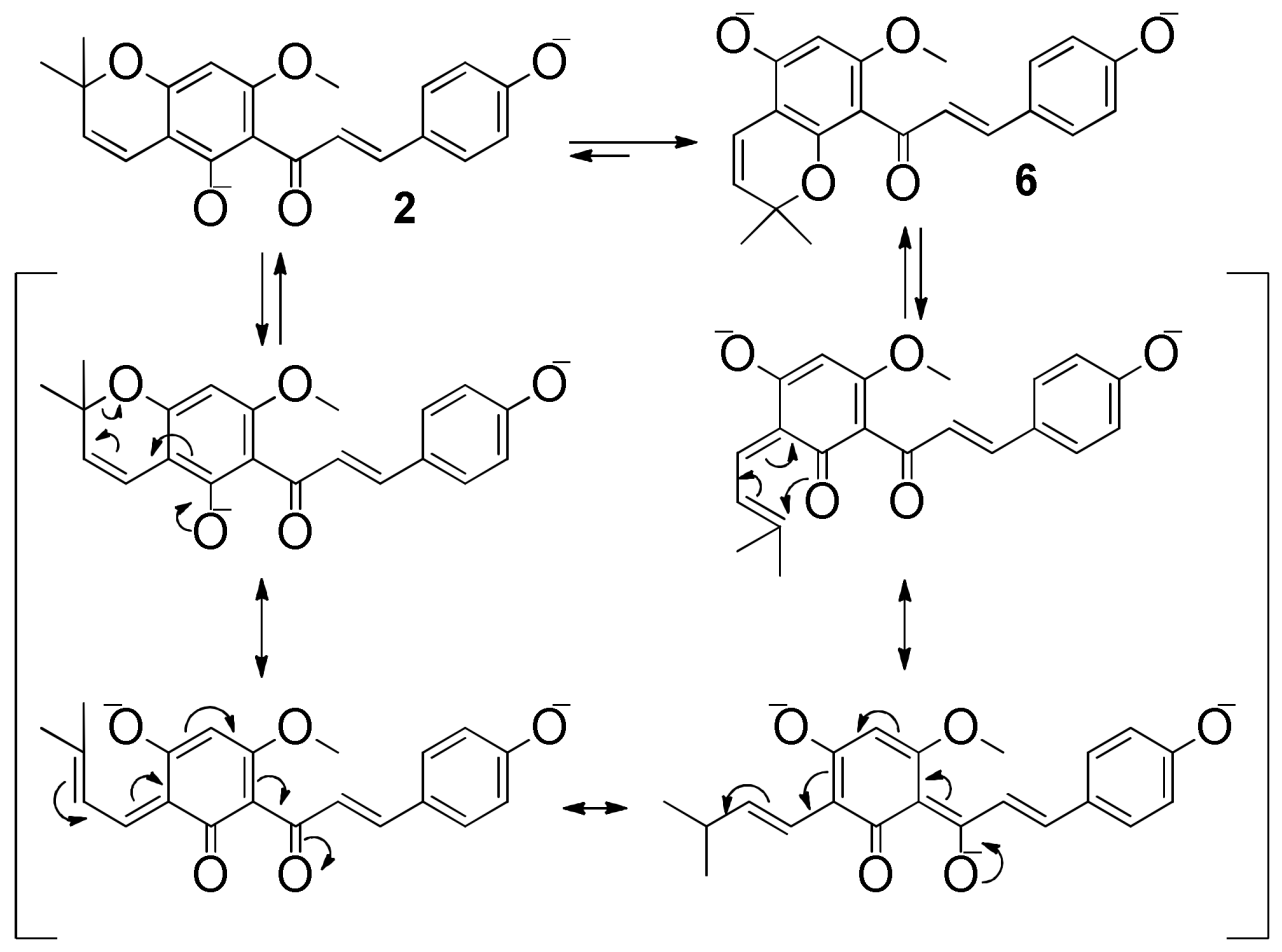
Proposed isomerization mechanism for the conversion of xanthohumol C (**2**) into xanthohumol K (**6**).

Further reaction under reflux conditions yielded xanthohumol K (**3**) in 66% yield. Since such a reaction was rather unexpected we decided to test it further with xanthohumol (**1**), xanthohumol K (**6**), 1″,2″-dihydroxanthohumol C (**3**) and 1″,2″-dihydroxanthohumol K (**4**). We thus obtained isoxanthohumol (**5**) from xanthohumol (**1**), and xanthohumol C (**2**) from xanthohumol K (**6**), whereas in case of **3** and **4** there were no reaction products. These results indicate the significance of the double bond of the dimethylchromene moiety for the piperidine-catalyzed isomerization between **2** and **6**. Furthermore it also indicates that compounds **2** and **3** are stable under aqueous base conditions and therefore possibly less prone to chalcone-flavanone isomerisation during in vitro assays. Interestingly, to the best of our knowledge there are no reports of such isomerizations of chromenones, therefore it is highly possible that such an isomerization is related only to chromenes with a carbonyl group in a position *ortho* to one of the reactive hydroxyl groups. Although this unusual isomerization requires more explanation, we would like to propose the reaction mechanism shown in Scheme 4 based on our observations. The first step involves deprotonation of hydroxyl groups and rearrangement of aromatic and chromene ring π-bonds resulting in an electrocyclic chromene ring opening. Subsequent rearrangement of the π-bond system, stabilized by the adjacent carbonyl group, enables prenyl group isomerization and further electrocyclic new chromene ring formation. Therefore the reaction seems to be an anionic variant of an oxy-Cope rearrangement, however further investigation is required to confirm the role of the carbonyl group and conjugated α,β-unsaturated bond-chalcone ring B system.

One of the commonly observed structure-activity relationships among flavonoids is the importance of the C2-C3 double bond positively influencing the biological activity of flavonoids with a heterocyclic ring C [36,37,38]. Although, no prenyl or geranyl flavones are detected in hops we decided to obtain 2,3-dehydroisoxanthohumol (**13**) and determine its antiproliferative activity. Among methods of direct oxidative cyclization of chalcones to flavones the most promising method is the reaction of the chalcone with iodine in DMSO [39]. However, such a reaction with **1**, despite the wide range of iodine amounts added to the reaction mixture (0.01–1 equiv.) resulted in a complex mixture of many chalcones and flavones, probably with iodine substituted carbons at C-5′ or prenyl group iodine addition reaction products. Therefore, another approach was to start from isoxanthohumol (**5**) and use an iodine-pyridine complex that should not lead to unwanted substitutions or additions. Reaction of **5** with I_2_-pyridine afforded **13** in 60% yield. Following our synthetic route for cyclized products we performed the reaction of **13** with AlCl_3_ in acetonitrile, because of the very limited solubility of **13** in DCM. The reaction gave **14** in 69% yield.

To compare the activity of compounds obtained here with our previous results concerning the antiproliferative properties of prenylated hops flavonoids and their fungal metabolites, we decided to use the same antiproliferative assay and the same three cancer cell lines: prostate (PC-3), colon (HT-29) and breast cancer (MCF-7) [23,40].

Antiproliferative potency was evaluated using the SRB assay and expressed as IC_50_ values, which are the concentration of a compound that inhibits the proliferation rate of tumor cells by 50% as compared to untreated control cells [41]. Four chalcones (**2**–**4**, **6**), two α,β-dihydrochalcone derivatives (**8**, **9**), three flavanones (**10**, **11a**, **11b**) and three flavones (**12**–**14**) have been subjected to this assay. Xanthohumol (**1**) and cisplatin were used as reference compounds. The results are presented in Table 2. All tested compounds exhibited moderate or strong antiproliferative activity wherein the most active was xanthohumol (**1**). The MCF-7 cell line is was the most vulnerable among the tested cell lines, which is in accordance with our previous reports [23,40].

Xanthohumol C (**2**) and 1″,2″-dihydroxanthohumol K (**4**) exhibited the strongest activity among the minor hop chalconoids, moreover, both of them are as active as cisplatin with respect to the PC-3 cell line. In contrast to xanthohumol (**1**), xanthohumols C (**2**) and K (**6**) were not the subject of a broader study of their anticancer activity. Studies by Miranda et al. have shown that **2** was slightly less active than **1** against MCF-7 and ovarian cancer cells A-278 [42]. While the antiproliferative activity of **2** in relation to the MCF-7 line was comparable to our results (MIC_50_ = 15.7 µM, after 2 days of exposure), the activity in relation to the HT-29 line was much weaker.

Xanthohumol C (**2**) has been also reported to suppress cancer invasion in relation to matrix metalloproteinase (MMP-2 and MMP-9) expression in HT-1080 human fibrosarcoma cells and inhibited angiogenesis by suppression of vascular endothelial growth factor (VEGF) [43]. This compound was proven to be the strongest agent promoting neurogenesis, neuroregeneration and neuroprotection among the seven tested hop flavonoids (**1**, **2**, **3**, **5**, isoxanthohumol C, 6- and 8-prenylnaringenin) [44].

It is noteworthy that saturation of **2** decreased the activity, coinciding with the results obtained by Vogel and Heilmann [15]. On the other hand, the saturation of the 1″,2″-double bond in **6** had the opposite effect and increased the antiproliferative activity. Saturation of the α,β-double bond in chalcones **3** and **4** resulted in a significant loss of the activity (except for the MCF-7 line and compound **8**) indicating that the increased activity of α,β-dihydroxanthohumol compared to xanthohumol (**1**), which was previously reported [23], is the exception rather than a rule.

Among the compounds obtained from xanthohumol (**1**), but not found in hops, the most active turned out to be 2,3-dehydroisoxanthohumol (**13**), which antiproliferative activities against PC-3 and MCF-7 lines were as high as cisplatin and against HT-29 line about twice lower than that of cisplatin. Comparing the results obtained with compounds **12**, **13** and **14** it can be assumed that the free prenyl group determines the high antiproliferative activity of the flavones tested.

## 3. Materials and Methods

### 3.1. General Information

All chemical reagents and organic solvents used for chemical synthesis were purchased from Merck (Darmstadt, Germany), Sigma-Aldrich, (Darmstadt, Germany) or Fluka (Darmstadt, Germany). TLC analysis was carried out on Merck silica gel 60, F254 (0.2 mm thick) plates with chloroform/methanol (9:1 *v*/*v*) or hexane/acetone (1:1 *v*/*v*) as eluents. HPLC was performed on a 2695 Alliance instrument (Waters, Milford, MA, USA) equipped with a Waters 2996 photodiode array detector using a Cosmosil Cholester 5 µm (4.6 × 250 mm) (Nacalai Tesque Inc. Kyoto, Japan), analytical HPLC column at a flow rate of 1 mL/min. A linear solvent gradient from 45% to 95% *aq* MeOH containing 0.05% HCOOH over 39 min was used. The products of reactions were separated by column chromatography on silica gel 60 (230–400 mesh, Merck) using chloroform, methanol, hexane or acetone mixtures as eluents. Quantitative analysis of TFA reactions was performed by means of HPLC (detection at 340 nm). NMR spectra (^1^H-NMR, ^13^C-NMR, COSY, HMQC, HMBC) were recorded on a DRX Avance™ 600 (600 MHz) instrument (Bruker, Billerica, MA, USA) in acetone-d*_6_*, methanol-d*_4_* or CDCl_3_. UV spectra were recorded on a Cintra 303 GBC spectrophotometer (GBC Scientific Equipment, Braeside, VIC, Australia) in methanol. Negative-ion HR-ESI-MS spectra were taken on a Bruker microTOF-Q instrument (Bruker, Billerica, MA, USA).

### 3.2. Antiproliferative Assay in Vitro

#### 3.2.1. Cells

The following established in vitro cancer cell lines were applied: MCF-7 (human breast carcinoma), PC-3 (human prostate cancer) and HT-29 (human colon cancer). All cancer cell lines were obtained from American Type Culture Collection (Rockville, MD, USA) and are being maintained in the Institute of Immunology and Experimental Therapy (Wroclaw, Poland). MCF-7 cells were cultured in Eagle medium supplemented with 2 mM l-glutamine and 10 mM sodium pyruvate, 10% fetal bovine serum and 0.8 mg/L of insulin (from Sigma–Aldrich Chemie GmbH, Steinheim, Germany). PC-3 cells were cultured in RPMI 1640 medium, supplemented with 2 mM l-glutamine, 10% fetal bovine serum (both from Sigma–Aldrich Chemie GmbH). HT-29 cells were cultured in mixture of OptiMEM and RPMI 1640 (1:1) medium (purchased from Gibco, Scotland, UK), supplemented with 2 mM l-glutamine (Sigma-Aldrich Chemie GmbH), 5% fetal bovine serum (Thermo Fisher Scientific, Waltham, MA USA) medium was additionally supplemented with 1 mM sodium pyruvate (Sigma-Aldrich Chemie GmbH). All culture media were also supplemented with antibiotics: 100 units/mL penicillin and 100 µg/mL streptomycin (purchased from Polfa Tarchomin SA, Warsaw, Poland). All cell lines were grown at 37 °C with 5% CO_2_ humidified atmosphere. Twenty-four hours before addition of tested agents, the cells were plated in 96-well plates (Sarstedt, Nümbrecht, Germany) at a density of 10^4^ cells per well in 100 µL of culture medium. An assay was performed after 72 h of exposure to varying concentrations of the tested agents (from 0.1 to 100 µg/mL). The results were calculated as the IC_50_ (inhibitory concentration 50%), the concentration of tested agent which inhibits 50% of the proliferation of the cancer cell population. IC_50_ values were calculated for each experiment separately and mean values ±SD are presented in the Table 2. Each compound at each concentration was tested in triplicate in a single experiment, which was repeated 3–5 times. Dimethyl sulphoxide, which was used as a solvent (in a dilution corresponding to its highest concentration applied to the tested compounds), did not exert any inhibitory effect on cell proliferation. To evaluate antiproliferative effect of tested compounds, the SRB method was applied.

#### 3.2.2. SRB Assay

The details of this technique were described by Skehan et al. [41]. The cells attached to the plastic were fixed by gently layering cold 50% trichloroacetic acid (TCA) (Sigma-Aldrich Chemie GmbH) on the top of the culture medium in each well. The plates were incubated at 4 °C for 1 h and then washed five times with tap water. The background optical density was measured in the wells filled with culture medium, without the cells. The cellular material fixed with TCA was stained with 0.4% sulforhodamine B (Sigma-Aldrich Chemie GmbH), dissolved in 1% acetic acid (POCh, Gliwice, Poland) for 30 min. Unbound dye was removed by rinsing (five times) with 1% acetic acid. The protein-bound dye was extracted with 150 µL of 10 mM unbuffered Tris base (Sigma-Aldrich Chemie GmbH) for determination of optical density (at 545 nm) in a computer-interfaced, 96-well microtiter plate reader (Synergy H4 Hybrid Reader, Biotek Instruments, Highland Park, VT, USA).

### 3.3. Chemical Synthesis

#### 3.3.1. Xanthohumol (**1**)

Xanthohumol (**1**) was isolated from supercritical carbon dioxide extracted hops (”Marynka”, crop 2012), obtained from Production of Hop Extracts (New Chemical Syntheses Institute, Puławy, Poland), following the method described by Bartmańska et al. [27]. Spectroscopic data of the isolated xanthohumol (**1**) were in agreement with literature values.

#### 3.3.2. Xanthohumol C (**2**)

DDQ (227 mg, 1 mmol) and **1** (354 mg, 1 mmol) were dissolved in anhydrous 1,4-dioxane (25 mL). The reaction mixture was heated to 95 °C for 4 h, then cooled to room temperature and 40 mL of ethyl acetate was added. The mixture was washed with equal volumes (20 mL) of water, saturated NaHCO_3_ solution, water and brine. The organic phase was dried over MgSO_4_, evaporated and purified by column chromatography (eluent: chloroform-methanol 9:1) to give xanthohumol C (**2**): yield 74%, orange amorphous powder; UV (MeOH) λ_max_ 233, 285, 370 nm; ^1^H-NMR (600 MHz, acetone-*d*_6_) δ: 1.42 (6H, s, H-4″, H-5″), 3.97 (3H, s, C6′-OCH_3_), 5.53 (1H, d, *J* = 9.9 Hz, H-2″), 6.00 (1H, s, H-5′), 6.62 (1H, d, *J* = 9.9 Hz, H-1″), 6.92 (2H, m, *J* = 7,1 Hz, H-3, H-5), 7.59 (2H, m, *J* = 7.1 Hz, H-2, H-6), 7.76 (1H, d, *J* = 15.4 Hz, H-β), 7.85 (1H, d, *J* = 15.4 Hz, H-α), and 14.85 (s, C2′-OH); ^13^C-NMR (150 MHz, acetone-*d*_6_) δ: 28.52 (C-4″, C-5″), 56.46 (C6′-OCH_3_), 78.73 (C-3″), 92.34 (C-5′), 103.36 (C-3′), 106.46 (C-1′), 116.53 (C-1″), 116.76 (C-3, C-5), 124.96 (H-α), 126.20 (C-2″), 127.87 (C-1), 131.30 (C-2, C-6), 143.71 (H-β), 160.66 (C-4), 161.01 (C-4′), 163.24 (C-2′), 163.64 (C-6′), and 193.32 (C=O). HR ESI-MS *m*/*z*: 351.1234 [M − H]^−^ (calcd for C_21_H_20_O_5_ − H, 351.1238).

#### 3.3.3. 1″,2″-Dihydroxanthohumol C (**3**)

A mixture of **1** (354 mg, 1 mmol), anhydrous AlCl_3_ (532 mg, 4 mmol) and methylene chloride (100 mL) was stirred at room temperature for 5 h. The reaction mixture was filtered, the filtrate washed with water (3 × 70 mL), dried over MgSO_4_, evaporated and purified by column chromatography (eluent: chloroform-methanol 9:1) to yield 1″,2″-dihydroxanthohumol C (**3**): yield 87%, orange amorphous powder; UV (MeOH) λ_max_ 370 nm; ^1^H-NMR (600 MHz, acetone-*d*_6_) δ: 1.34 (6H, s, H-4″, H-5″), 1.81 (2H, t, *J* = 6.8 Hz, H-2″), 2.57 (2H, t, *J* = 6.8 Hz, H-1″), 3.95 (3H, s, C6′-OCH_3_), 5,93 (1H, s, H-5′), 6.92 (2H, m, *J* = 8.6 Hz, H-3, H-5), 7.60 (2H, m, *J* = 8.6 Hz, H-2, H-6), 7.76 (1H, d, *J* = 15.5 Hz, H-α), 7.91 (1H, d, *J* = 15.5 Hz, H-β) and 14.93 (s, C2′-OH); ^13^C-NMR (600 MHz, acetone-*d*_6_) δ: 16.77 (C-1″), 26.90 (C-4″, C-5″), 32.60 (C-2″), 56.22 (C6′-OCH_3_), 76.68 (C-3″), 92.59 (C-5′), 102.47 (C-3′), 105.97 (C-1′), 116.79 (C-3, C-5), 125.26 (C-α), 128.07 (C-1), 131.25 (C-2, C-6), 143.31 (C-β), 160.60 (C-4′), 161.72 (C-2′), 161.75 (C-6′), 166.41 (C-4), 193.16 (C=O); HR ESI-MS *m*/*z*: 353.1391 [M − H]^−^ (calcd for C_21_H_22_O_5_ − H, 353.1394).

#### 3.3.4. 1″,2″-Dihydroxanthohumol K (**4**)

To a solution of **1** (354 mg, 1 mmol) in 99 mL of methylene chloride 1 mL of TFA was added dropwise (the addition over 5 min) and the reaction mixture was stirred at room temperature for 5 h. The reaction was stopped by a slow addition of saturated NaHCO_3_ while stirring the reaction mixture vigorously, until its color changed to yellow. Then the organic phase was washed with water (3 × 70 mL), dried over MgSO_4_, evaporated and purified by column chromatography (eluent: chloroform-methanol 9:1) to afford 1″,2″-dihydroxanthohumol K (**4**): yield 71%, orange amorphous powder; UV (MeOH) λ_max_ 331 nm; ^1^H-NMR (600 MHz, acetone-*d*_6_) δ: 1.22 (6H, s, H-4″, H-5″), 1.75 (2H, t, *J* = 6.7 Hz, H-2″), 2.63 (2H, t, *J* = 6.7 Hz, H-1″), 3.62 (3H, s, C6′-OCH_3_), 6.19 (1H, s, H-5′), 6.77 (1H, d, *J* = 16.0 Hz, H-α), 6.88 (2H, m, *J* = 8.6 Hz, H-3, H-5), 7.23 (1H, d, *J* = 16.0 Hz, H-β), 7.48 (2H, m, *J* = 8.6 Hz, H-2, H-6); ^13^C-NMR (150 MHz, acetone-*d*_6_) δ: 17.39 (C-1″), 26.80 (C-4″, C5″), 32.61 (C-2″), 55.87 (C6′-OCH_3_), 75.07 (C-3″), 92.01 (C-5′), 101.98 (C-3′), 111.52 (C-1′), 116.72 (C-3, C-5), 127.52 (C-1), 127.62 (C-α), 130.82 (C-2, C-6), 144.26 (C-β), 153.72 (C-2′), 157.41 (C-6′), 157.56 (C-4′), 160.42 (C-4), 194.46 (C=O); HR ESI-MS *m*/*z*: 353.1392 [M − H]^−^ (calcd for C_21_H_22_O_5_ − H, 353.1394).

#### 3.3.5. Isoxanthohumol (**5**)

Isoxanthohumol (**5**) was obtained from **1** by base catalyzed isomerization following the method described by Bartmańska et al. [27]. Spectroscopic data of the obtained isoxanthohumol (**5**) were in agreement with literature values.

#### 3.3.6. Xanthohumol K (**6**)

DDQ (45.4mg, 0.2 mmol) and **4** (70.8 mg, 0.2 mmol) were dissolved in anhydrous 1,4-dioxane (25 mL). The reaction mixture was heated to 95 °C for 4 h, then cooled to room temperature and 40 mL of ethyl acetate was added. The reaction mixture was washed with equal volumes (20 mL) of water, NaHCO_3_, water and brine. The organic phase was dried over MgSO_4_, evaporated and subjected to column chromatography (eluent: hexane-acetone 1:1) to give xanthohumol K (**6**): yield 17% and 66%, yellow-orange amorphous powder; UV (MeOH) λ_max_ 288, 331 nm; ^1^H-NMR (600 MHz, acetone-*d*_6_) δ: 1.31 (6H, s, H-4″, H-5″), 3.67 (3H, s, C6′-OCH_3_), 5.52 (1H, d, *J* = 9.9 Hz, H-2″), 6.17 (1H, s, H-5′), 6.64 (1H, d, *J* = 9.9 Hz, H-1″), 6.77 (1H, d, *J* = 16.0 Hz, H-β), 6.88 (2H, m, *J* = 8.6 Hz, H-3, H-5), 7.26 (1H, d, *J* = 16.0 Hz, H-α), 7.52 (2H, m, *J* = 8.6 Hz, H-2,H-6); ^13^C-NMR (150 MHz, acetone-*d*_6_) δ: 28.03 (C-4″, C-5″), 55.98 (C6′-OCH_3_), 77.12 (C-3″), 92.86 (C-5′), 103.92 (C-3′), 112.15 (C-1′), 116.80 (C-3, C-5), 117.56 (C-1″), 127.03 (C-2″), 127.57 (C-1), 127.65 (H-α), 130.96 (C-2, C-6), 144.65 (H-β), 152.86 (C-2′), 155.20 (C-6′), 159.05 (C-4′), 160.55 (C-4), 193.62 (C=O); HR ESI-MS *m*/*z*: 351.1236 [M − H]^−^ (calcd for C_21_H_20_O_5_ − H, 351.1238).

Alternatively, a distilled water (10 mL) piperidine was added (0.1 mL) followed by **2** (70.4 mg, 0.2 mmol). The reaction mixture was refluxed for 20 min, then cooled to room temperature, neutralized with 1M HCl and extracted with ethyl acetate (3 x 20 mL). The organic phase was dried over MgSO_4_, evaporated and subjected to column chromatography (eluent: hexane-acetone 1:1) to give compound **6**.

#### 3.3.7. 4.4′-Dimethoxymethyl xanthohumol (**7**)

To a mixture of **1** (70.8 mg, 0.2 mmol) and anhydrous K_2_CO_3_ (96.6 mg, 0.35 mmol) in acetone (5 mL) MOMCl (180 µL, 4.6 mmol) was added dropwise at 0 °C. The reaction mixture was stirred at room temperature for 3 h, then filtered and evaporated. The residue was subjected to column chromatography using hexane-acetone 1:1 as eluent to afford the intermediate 4.4′-dimethoxymethyl xanthohumol (**7**): yield 85%. yellow-orange oil; UV (MeOH) λ_max_ 366 nm; ^1^H-NMR: (600 MHz, CDCl_3_) δ: 1.67 (3H, s, H-5′’), 1.79 (3H, s, H-4″), 3.32 (2H, d, *J* = 7.2 Hz, H-1″), 3.48 (3H, s, MOM-CH_3_), 3.49 (3H, s, MOM-CH_3_), 3.91 (3H, s, C6′-OCH_3_), 5.21 (2H, s, MOM-CH_2_), 5.22 (1H, m, H-2″), 5.26 (2H, s, MOM-CH_2_), 6.23 (1H, s, H-5′), 7.05 (2H, m, *J* = 8.8 Hz, H-3, H-5), 7.54 (2H, m, *J* = 8.8 Hz, H-2, H-6), 7.73 (1H, d, *J* = 15.7 Hz, H-α), 7.79 (1H, d, *J* = 15.7 Hz, H-β) and 14.12 (s, C2′-OH).

#### 3.3.8. 1″,2″,α,β-Tetrahydroxanthohumol C (**8**)

A mixture of **3** (35.4 mg, 0.1 mmol) and 5% Pd/C (70.8 mg, 2 weight equiv.) in 10 mL of methanol was stirred at room temperature. The mixture was bubbled with nitrogen for 10 min, then with hydrogen from a balloon for another 10 min and then stirred for the next 5 min. The reaction was filtered, evaporated and purified by column chromatography (eluent: hexane-acetone 1:1) to yield 1″,2″,α,β-tetrahydroxanthohumol C (**8**): yield 91%, white amorphous powder; UV (MeOH) λ_max_ 295 nm; ^1^H-NMR (600 MHz, acetone-*d*_6_) δ: 1.32 (6H, s, H-4″, H-5″), 1.80 (2H, t, *J* = 6.8 Hz, H-2″), 2.55 (2H, t, *J* = 6.8 Hz, H-1″), 2.85 (2H, m, H-β), 3.26 (2H, m, H-α), 3.87 (3H, s, C6′-OCH_3_), 5.91 (1H, s, H-5′), 6.76 (2H, m, *J* = 8.4 Hz, H-3, H-5), 7.09 (2H, m, *J* = 8.4 Hz, H-2, H-6), and 14.43 (s, C2′-OH); ^13^C-NMR (150 MHz, acetone-*d*_6_) δ: 16.71 (C-1″), 26.86 (C-4″, C-5″), 30.78 (H-β), 32.57 (C-2″), 46.69 (H-α), 56.08 (C6-CH_3_), 76.68 (C-3″), 92.36 (C-5′), 102.32 (C-3′), 105.48 (C-1′), 116.02 (C-3, C-5), 130.18 (C-2, C-6), 133.33 (C-1), 156.47 (C-4), 161.70 (C-4′), 162.02 (C-6′), 165.60 (C-2′), and 205.55 (C=O); HR ESI-MS *m*/*z*: 355.1550 [M − H]^−^ (calcd for C_21_H_24_O_5_ − H, 355.1551).

#### 3.3.9. 1″,2″,α,β-Tetrahydroxanthohumol K (**9**)

1″,2″,α,β-Tetrahydroxanthohumol K (**9**) was obtained according to the same procedure as **8**, using **4** as a substrate. Yield 88%, white amorphous powder; UV (MeOH) λ_max_ 278 nm; ^1^H-NMR (600 MHz, acetone-*d*_6_) δ: 1.26 (6H, s, H-4″, H-5″), 1.76 (3H, t, *J* = 6.9 Hz, H-2″), 2.60 (2H, t, *J* = 6.9 Hz, H-1″), 2.82 (2H, m, H-β), 2.91 (2H, m, H-α), and 3.65 (3H, s, C6′-OCH_3_), 6.12 (1H, s, H-5′), 6.73 (2H, m, *J* = 8.4 Hz, H-3, H-5), 7.05 (2H, m, *J* = 8.4 Hz, H-2, H-6); ^13^C-NMR (150 MHz, acetone-*d*_6_) δ: 17.32 (C-1″), 26.80 (C-4″, C-5″), 29.82 (C-β), 32.63 (C-2″), 47.77 (C-α), 55.92 (C6′-OCH_3_), 75.30 (C-5″), 91.93 (C-5′), 102.01 (C-3′), 113.42 (C-1′), 115.92 (C-3, C-5), 130.04 (C-2, C-6), 133.41 (C-1), 153.15 (C-2′), 156.34 (C-6′), 156.68 (C-4), 157.46 (C-4′), and 202.63 (C=O); HR ESI-MS *m*/*z*: 355.1548 [M − H]^−^ (calcd for C_21_H_24_O_5_ − H, 355.1551).

#### 3.3.10. 1″,2″-Dihydroisoxanthohumol C (**10**)

A mixture of **5** (354 mg, 1 mmol), anhydrous AlCl_3_ (532 mg, 4 mmol) and methylene chloride (100 mL) was stirred at room temperature for 5 h. The reaction mixture was filtered, the filtrate washed with water (3 × 70 mL), dried over MgSO_4_, evaporated and purified by column chromatography (eluent: chloroform-methanol 9:1) to yield 1″,2″-dihydroisoxanthohumol C (**10**): yield 76%, beige amorphous powder; UV (MeOH) λ_max_ 289 nm; ^1^H-NMR (600 MHz, acetone-*d*_6_) δ: 1.31 (3H, s, H-4″), 1.33 (3H, s, H-5″), 1.79 (2H, m, H-2″), 2.57 (2H, m, H-1″), 2.62 (1H, dd, *J* = 2.9 Hz, 16.2 Hz, H-3, *eq*), 2.93 (1H, dd, *J* = 12.9 Hz, 16.2 Hz, H-3, *ax*), 3.77 (3H, s, C5-OCH_3_), 5.40 (1H, dd, *J* = 2.9 Hz, 12.9 Hz, H-2), 6.00 (1H, s, H-6), 6.90 (2H, m, *J* = 8.5 Hz, H-3′, H-5′), 7.40 (2H, m, *J* = 8.5 Hz, H-2′, H-6′); ^13^C-NMR (600 MHz, acetone-*d*_6_) δ: 17.28 (C-1″), 26.58 (C-4″), 27.16 (C-5″), 32.60 (C-2″), 46.09 (C-3), 55.92 (C5-OCH_3_), 76.33 ( C-3″), 79.56 (C-2), 94.62 (C-6), 102.34 (C-8), 106.10 (C-10), 116.06 (C-3′, C-5′), 128.62 (C-2′, C-6′), 131.48 (C-1′), 158.34 (C-4′), 160.95 (C-7), 161.14 (C-5), 162.54 (C-9), 188.40 (C=O); HR ESI-MS *m*/*z*: 353.1394 [M − H]^−^ (calcd for C_21_H_24_O_5_ − H, 353.1394)).

#### 3.3.11. 5,4′-Dihydroxy-6″,6″-dimethyl-4″,5″-dihydropyrano-[2″,3″:7,8]flavanone (**11a**) and 5,4′-dihydroxy-6″,6″-dimethyl-4″,5″-dihydropyrano-[2″,3″:6,7]flavanone (**11b**)

A mixture of **5** (177 mg, 0.5 mmol), anhydrous AlCl_3_ (266 mg, 2 mmol) and anhydrous acetonitrile (50 mL) was stirred at 50 °C for 24 h. The reaction mixture was filtered, the filtrate washed with water (3 × 30 mL), dried over MgSO_4_, evaporated and purified by column chromatography (eluent: chloroform-methanol 9:1) to yield a mixture of 5,4'-dihydroxy-6″,6″- dimethyl-4″,5″-dihydropyrano-[2″,3″:7,8]flavanone (**11a**) and 5,4'-dihydroxy-6″,6″-dimethyl-4″,5″- dihydropyrano-[2″,3″:6,7]flavanone (**11b**). yield 80%, cream amorphous powder; UV (MeOH) λ_max_ 293, 333 nm; ^1^H-NMR (600 MHz, acetone-*d*_6_) δ: 1.31 (3H, s, H-4″), 1.33 (3H, s, H-5″), 1.79 (2H, m, H-2″), 2.56 (2H, m, H-1″), 2.77 (1H, dd, *J* = 3.0 Hz, 16.9 Hz, H-3, *eq*), 3.16 (1H, ddd, *J* = 1.36, 12.9 Hz, 16.9 Hz, H-3a, H-3b, *ax*), 5.50 (1H, dd, *J* = 3.0 Hz, 12.9 Hz, H-2), 5.83 (1H, s, H-6), 6.91 (2H, m, *J* = 8.5 Hz, H-3′, H-5′), 7.42 (2H, m, *J* = 8.5 Hz, H-2′, H-6′), 11.88 (s, C5-OH); ^13^C-NMR (600 MHz, acetone-*d*_6_) δ: 16.94 (C-1″), 26.56 (C-4″), 27.22 (C-5″), 32.39 (C-2″), 43.34 (C-3a), 43.39 (C-3b), 76.82 (C-3″), 79.87 (C-2), 97.43 (C-6a), 97.50 (C-6b), 101.51 (C-8a), 101.54 (C-8b), 103.24 (C-10a), 103.30 (C-10b), 116.13 (C-3′a, C- 5′a),116.21 (C-3′b, C-5′b), 128.86 (C-2′, C-6′), 130.93 (C-1′), 158.56 (C-4′a), 158.66 (C- 4′b), 161.10 (C-7a), 161.12 (C-7b), 162.13 (C-9a), 162.39 (C-9b), 163.53 (C-5a), 163.58 (C- 5b), 197.40 (C=O a), 197.48 (C=O b); HR ESI-MS *m*/*z*: 339.1234[M − H]^−^ (calcd for C_20_H_20_O_5_ − H, 339.1238).

#### 3.3.12. 2,3-Dehydroisoxanthohumol C (**12**)

DDQ (45.4 mg, 2 equiv.) and **5** (35.4 mg, 1 equiv.) were dissolved in anhydrous 1,4-dioxane (10 mL). The reaction mixture was heated to 95 °C for 4 h, then cooled to room temperature and 40 mL of ethyl acetate was added. The reaction mixture was washed with equal volumes (20 mL) of water, NaHCO_3_, water and brine. The organic phase was dried over MgSO_4_, evaporated and subjected to column chromatography (eluent: hexane-acetone 1:1) to give 2,3-dehydroisoxanthohumol C (**12**): yield 76%, yellow amorphous powder; UV (MeOH) λ_max_ 238, 289, 335 nm; ^1^H-NMR (600 MHz, acetone-*d*_6_) δ 1.48 (6H, s, H-4″, H-5″), 3.88 (3H, s, C5-OCH_3_), 5.77 (2H, d, *J* = 10.0 Hz, H-2″), 6.39 (1H, s, H-6), 6.47 (1H, s, H-3), 6.95 (2H, d, *J* = 10.0 Hz, H-1″) 7.03 (2H, m, *J* = 8.8 Hz, H-3′, H-5′), 7.90 (2H, m, *J* = 8.8 Hz, H-2′, H-6′); ^13^C-NMR (600 MHz, acetone-*d*_6_) δ: 28.30 (C-4″, C-5″), 56.49 (C5-OCH_3_), 78.65 (C-3″), 97.41 (C-6), 103.45 (C-8), 107.45 (C-3), 109.55 (C-10), 116.75 (C-1″), 116.84 (C-3′, C-5′), 123.64 (C-1′), 128.63 (C-2″), 128.64 (C-2′, C-6′), 154.61 (C-9), 158.46 (C-7), 160.95 (C-2), 161.32 (C-4′), 161.58 (C-5), 176.48 (C=O); HR ESI-MS *m*/*z*: 349.1080 [M − H]^−^ (calcd for C_21_H_18_O_5_ − H, 349.1081).

#### 3.3.13. 2,3-Dehydroisoxanthohumol (**13**)

Iodine (38 mg, 0.3 mmol) was dissolved in 10 mL of anhydrous pyridine and the mixture was stirred at room temperature, after 1 h isoxanthohumol (**5**) (106 mg, 0.3 mmol) was added and the mixture was stirred at 90 °C for 3h. The reaction was cooled and stopped by adding 10 mL of water and 3 mL of saturated Na_2_S_2_O_3_ solution, then neutralized with 1 M HCl solution and extracted with ethyl acetate (3 × 40 mL), dried over MgSO_4_, evaporated and purified by column chromatography (eluent: chloroform-methanol 5:1) to yield 2,3-dehydroisoxanthohumol (**13**): yield 60%, white-yellow amorphous powder; UV (MeOH) λ_max_ 268, 335 nm; ^1^H-NMR (600 MHz, metanol-*d*_4_) δ 1.67 (3H, s, H-4″), 1.81 (3H, s, H-5″), 3.54 (2H, d, *J* = 6.7 Hz, H-1″), 3.86 (1H, s, C5-OCH_3_), 5.23 (1H, t, *J* = 6.7 Hz, H-2″), 6.43(1H, s, H-6), 6.52 (1H, s, H-3), 6.91 (2H, m, *J* = 8.8 Hz, H-3′, H-5′), 7.78 (2H, m, *J* = 8.8 Hz, H-2′, H-6′); ^13^C-NMR (150 MHz, metanol-*d*_4_) δ: 18. 24 (C-5″), 22.71 (C-1″), 25.92 (C-4″), 56.09 (C5-OCH_3_), 96.60 (C-6), 106.04 (C-3), 108.09 (C-10), 109.32 (C-8), 116.67 (C-3′, C-5′), 123.36 (C-1′), 123.43 (C-2″), 128.81 (C-2′, C-6′), 132.51 (C-3″), 158.23 (C-9), 159.57 (C-5), 161.67 (C-7), 161.68 (C-4′), 163.29 (C-2), 180.58 (C=O); HR ESI-MS *m*/*z*: 351.1237 [M − H]^−^ (calcd for C_21_H_20_O_5_ − H, 351.1238).

#### 3.3.14. 1″,2″-Dihydro-2,3-dehydroisoxanthohumol C (**14**)

A mixture of **13** (35 mg, 0.1 mmol), anhydrous AlCl_3_ (53 mg, 0.4 mmol) and anhydrous acetonitrile (20 mL) was stirred at room temperature for 2 h. The reaction mixture was filtered, the filtrate washed with water (3 × 10 mL), dried over MgSO_4_, evaporated and purified by column chromatography (eluent: chloroform-methanol 5:1) to yield 1″,2″-dihydro-2,3-dehydroiso- xanthohumol C (**14**): yield 69%, yellow amorphous powder; UV (MeOH) λ_max_ 271, 335 nm; ^1^H-NMR (600 MHz, metanol-*d*_4_) δ: 1.40 (6H, s, H-4″, H-5″), 1.95 (2H, m, H-2″), 2.96 (2H, m, H-1″), 3.85 (3H, s, C5-OCH_3_), 6.39 (1H, s, H-6), 6.59 (1H, s, H-3), 6.94 (2H, m, *J* = 8.8 Hz, H-3′, H-5′), 7.85 (2H, m, *J* = 8.8 Hz, H-2′, H-6′); ^13^C-NMR (150 MHz, metanol-*d*_4_) δ: 17.59 (C-1″), 26.86 (C-4″, C-5″), 32.76 (C-2″), 56.35 (C5-OCH_3_), 77.43 (C-3″), 98.33 (C-6), 103.03 (C-8), 106.33 (C-3), 108.74 (C-10), 117.04 (C-3′, C-5′), 123.41 (C-1′), 129.07 (C-2′, C-6′), 152.97 (C-9), 158.69 (C-5), 160.09 (C-7), 160.75 (C-4′), 163.48 (C-2), 180.49 (C=O); HR ESI-MS *m*/*z*: 351.1239 [M − H]^−^ (calcd for C_21_H_20_O_5_ − H, 351.1238).

Supplementary materials contain all obtained ^1^H-NMR and ^13^C-NMR spectra of compounds presented in the manuscript.

#### 3.3.15. TFA Catalyzed Cyclisation of xanthohumol (**1**) with Various Catalyst Concentration and at Different Temperatures

Test reactions from Table 1 were performed in accordance to the synthesis of **4**, described in Section 3.3.4. There were differences in temperature and TFA amounts, as noted in Table 1. Also, the reactions were carried out in a smaller scale, using less substrate **1** (35.4 mg), smaller solvent volume (10 mL) and shorter reaction time (3 h). In each experiment after three hours 10 mL of water was added to the reaction mixture, while stirring vigorously. The organic phase was separated and dried over MgSO_4_. Then 0.1 mL of the organic phase was mixed with 1 mL of HPLC grade MeOH and analyzed by HPLC.

## 4. Conclusions

The results of in vitro tests indicate that chromane- and chromene-like cyclic prenylchalconoids are potent antiproliferative agents. These observations as well as the fact that some of the compounds obtained (**2**, **3**, **4**) arise as a result of metabolism in mammalian systems justify our research and further study on the pharmacological properties of hop compounds [45]. The most promising seem to be the minor hops prenlylchalconoids xanthohumol C (**2**) and 1″,2″-dihydroxanthohumol K (**4**) and non-natural 2,3-dehydroisoxanthohumol (**13**), which exhibited activity comparable to cisplatin. With high probability, these compounds will not be metabolized by gastrointestinal microbiome like xanthohumol (**1**) to 8-prenylnaringenin, which besides its assumed beneficial activities may promote mammary and endometrial cancers [46]. The development of efficient regioselective xanthohumol (**1**) prenyl group cyclisation reaction to give 1″,2″-dihydroxantohumol K (**4**) may be further applied in the synthesis of other compounds with the chromane moiety.

## Figures and Tables

**Table 1:** Cyclisation of xanthohumol (**1**) with TFA under different reaction conditions ^1^.

Temperature ^2^					TFA Amount ^3^				
°C	% ^4^ of 1	% ^4^ of 3	% ^4^ of 4	4/3	% (*v*/*v*)	% ^4^ of 1	% ^4^ of 3	% ^4^ of 4	4/3
0	54.6	9.5	35.9	3.8	0.1	97.5	0.4	2.1	5.3
22	16.7	16.7	66.6	4.0	0.5	46.5	9.8	43.7	4.5
39	0	18.7	81.3	4.3	1	10.3	16.8	72.9	4.3

^1^ The reaction conditions are specified in the Materials and Methods section. ^2^ Reactions with 0.8 % (*v*/*v*) of TFA. ^3^ Reactions performed at room temperature. ^4^ Conversions determined by HPLC.

**Table 2:** In vitro antiproliferative activity of xanthohumol (**1**) and synthetized compounds against human cancer cell lines.

Compound		Cancer Cell Line, IC_50_ (µM)		
		PC-3	HT-29	MCF-7
**1**	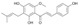	7.0 ± 1.5	10.1 ± 1.1	8.1 ± 0.8
**2**	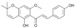	10.1 ± 3.0	10.6 ± 0.6	15.0 ± 1.8
**3**	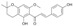	49.6 ± 6.6	16.7 ± 6.9	15.9 ± 3.6
**4**	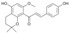	10.7 ± 5.9	12.5 ± 1.5	9.0 ± 6.4
**6**	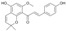	74.9 ± 25.6	87.5 ± 3.2	83.8 ± 14.6
**8**	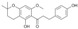	59.9 ± 5.4	62.7 ± 13.2	16.2 ± 2.1
**9**	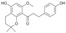	68.0 ± 14.2	89.1 ± 3.2	36.0 ± 3.6
**10**	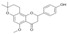	51.3 ± 21.2	85.4 ± 2.1	35.3 ± 6.6
**11a/11b**	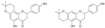	48.9 ± 7.8	25.8 ± 2.9	13.2 ± 3.7
**12**	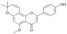	202.0 ± 0.0	217.1 ± 40.9	120.1 ± 3.6
**13**	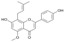	13.8 ± 1.4	20.1 ± 1.5	7.9 ± 0.5
**14**	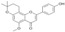	64.6 ± 10.7	91.2 ± 17.2	19.3 ± 6.1
cisplatin (reference)		12.33 ± 2.77	9.10 ± 1.13	8.27 ± 2.10

## Supplementary Materials

The following are available online. Supplementary materials contain all obtained ^1^H-NMR and ^13^C-NMR spectra of compounds presented in the manuscript.
