# Supplementary material for: Synthesis and Antiproliferative Activity of Minor Hops Prenylflavonoids and New Insights on Prenyl Group Cyclization
Source: Molecules. 2018 Mar 28;23(4):776. doi: 10.3390/molecules23040776 (PMC6017146; doi:10.3390/molecules23040776)
Supplement: Supplementary file 1 [file molecules-23-00776-s001.pdf]

Synthesis and antiproliferative activity of hops minor prenylflavonoids and new insights on prenyl group cyclization

SUPPLEMENTARY MATERIALS

Jarosław Popłoński[1]*, Eliza Turlej[2], Sandra Sordon[1], Tomasz Tronina[1], Agnieszka Bartmańska[1], Joanna Wietrzyk[2], Ewa Huszcza[2]

[1] Department of Chemistry, Wrocław University of Environmental and Life Sciences, Norwida 25, 50-375 Wrocław, Poland, jaroslaw.poplonski@upwr.edu.pl
[2] NeoLek Laboratory of Experimental Anticancer Therapy, Ludwik Hirszfeld Institute of Immunology and Experimental Therapy, Polish Academy of Sciences, Department of Experimental Oncology, Weigla 12, 53-114 Wrocław, Poland, wietrzyk@iitd.pan.wroc.pl
* Correspondence: jaroslaw.poplonski@upwr.edu.pl; Tel.: +48-71-320-5197

List of content:

1. ^{1}H NMR Spectrum of **2** - xanthohumol C.
2. ^{13}C NMR Spectrum of **2 -** xanthohumol C.
3. ^{1}H NMR Spectrum of **3** – 1’’,2’’-dihydroxanthohumol C.
4. ^{13}C NMR Spectrum of **3 -** 1’’,2’’-dihydroxanthohumol C.
5. ^{1}H NMR Spectrum of **4** – 1’’,2’’-dihydroxanthohumol K.
6. ^{13}C NMR Spectrum of **4 -** 1’’,2’’-dihydroxanthohumol K.
7. ^{1}H NMR Spectrum of **6** - xanthohumol K.
8. ^{13}C NMR Spectrum of **6 -** xanthohumol K.
9. ^{1}H NMR Spectrum of **7** - 4.4’-dimethoxymethyl xanthohumol
10. ^{1}H NMR Spectrum of **8** – 1’’,2’’,α,β-tetrahydroxanthohumol C.
11. ^{13}C NMR Spectrum of **8 -** 1’’,2’’,α,β-tetrahydroxanthohumol C.
12. ^{1}H NMR Spectrum of **9** – 1’’,2’’,α,β-tetrahydroxanthohumol K.
13. ^{13}C NMR Spectrum of **9 -** 1’’,2’’,α,β-tetrahydroxanthohumol K.
14. ^{1}H NMR Spectrum of **10** -1”,2”-Dihydroisoxanthohumol C.
15. ^{13}C NMR Spectrum of **10** -1”,2”-Dihydroisoxanthohumol C.
16. ^{1}H NMR Spectrum of **11a/11b -** 5,4'-dihydroxy-6",6"-dimethyl-4",5"-dihydropyrano-[2",3":7,8]flavanone and 5,4'-dihydroxy-6",6"-dimethyl-4",5"-dihydropyrano-[2",3":6,7]flavanone.
17. ^{13}C NMR Spectrum of **11a/11b** - 5,4'-dihydroxy-6",6"-dimethyl-4",5"-dihydropyrano-[2",3":7,8]flavanone and 5,4'-dihydroxy-6",6"-dimethyl-4",5"-dihydropyrano-[2",3":6,7]flavanone.
18. ^{1}H NMR Spectrum of **12 -** 2,3-Dehydroisoxanthohumol C.
19. ^{13}C NMR Spectrum of **12** - 2,3-Dehydroisoxanthohumol C.
20. ^{1}H NMR Spectrum of **13 -** 2,3-Dehydroisoxanthohumol.
21. ^{13}C NMR Spectrum of **13 -** 2,3-Dehydroisoxanthohumol.
22. ^{1}H NMR Spectrum of **14** - 1’’,2’’-Dihydro-2,3-dehydroisoxanthohumol C.
23. ^{13}C NMR Spectrum of **14 -** 1’’,2’’-Dihydro-2,3-dehydroisoxanthohumol C.

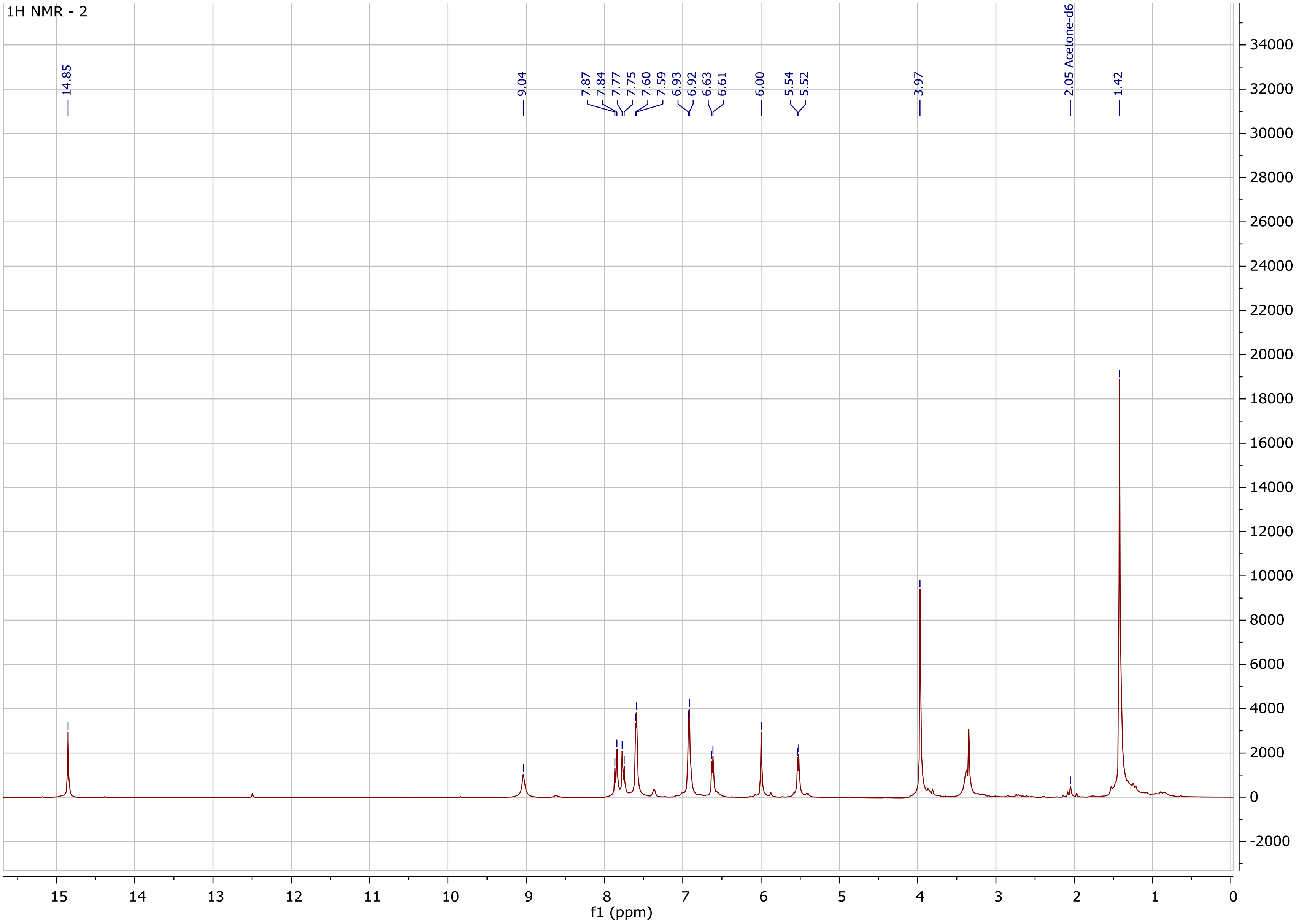
1H NMR - 2
14.85
9.04
7.87
7.84
7.77
7.75
7.60
7.59
6.93
6.92
6.63
6.61
6.00
5.54
5.52
3.97
2.05 Acetone-d6
1.42
f1 (ppm)

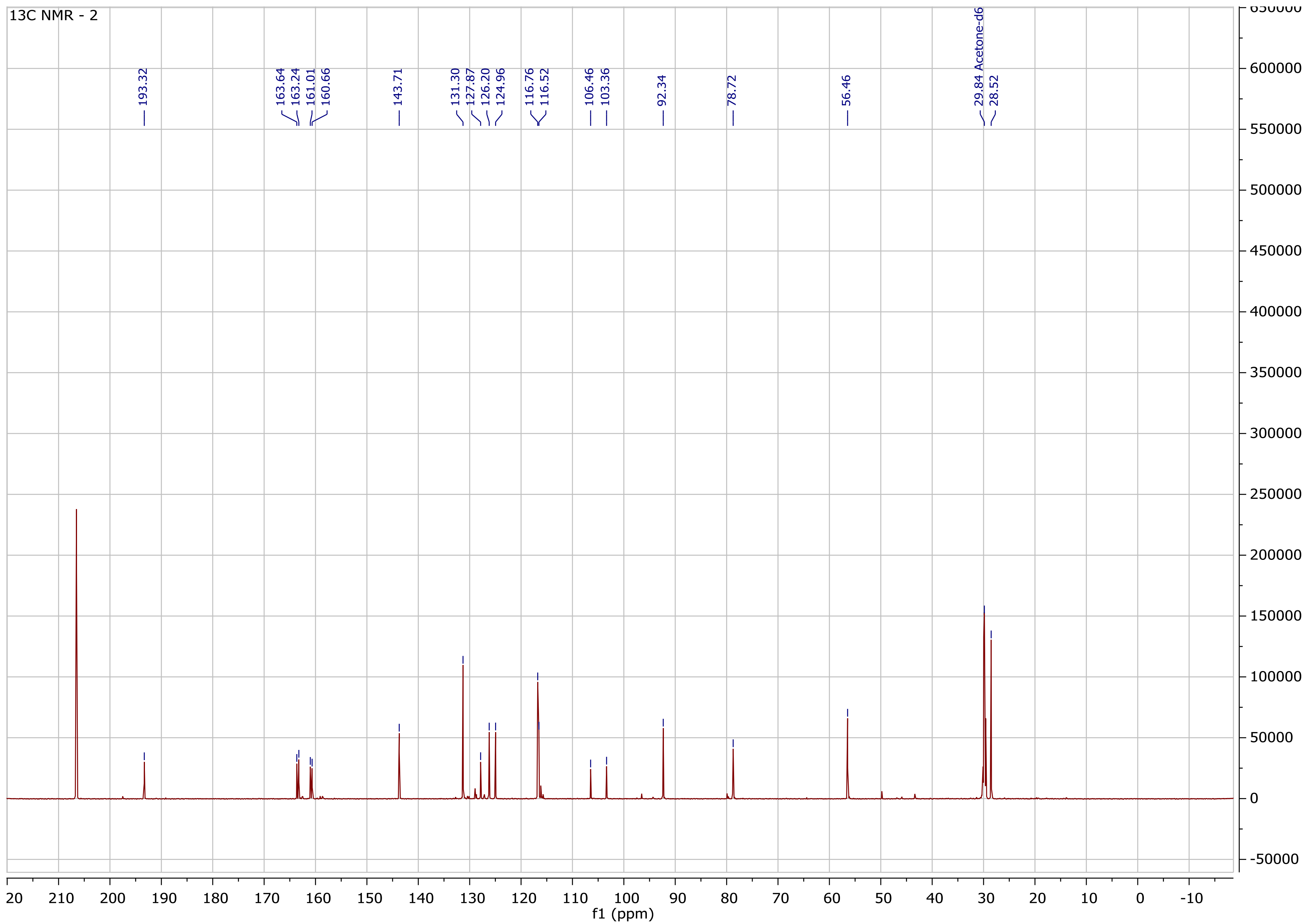
13C NMR - 2
193.32
163.64
163.24
161.01
160.66
143.71
131.30
127.87
126.20
124.96
116.76
116.52
106.46
103.36
92.34
78.72
56.46
29.84 Acetone-d6
28.52
f1 (ppm)

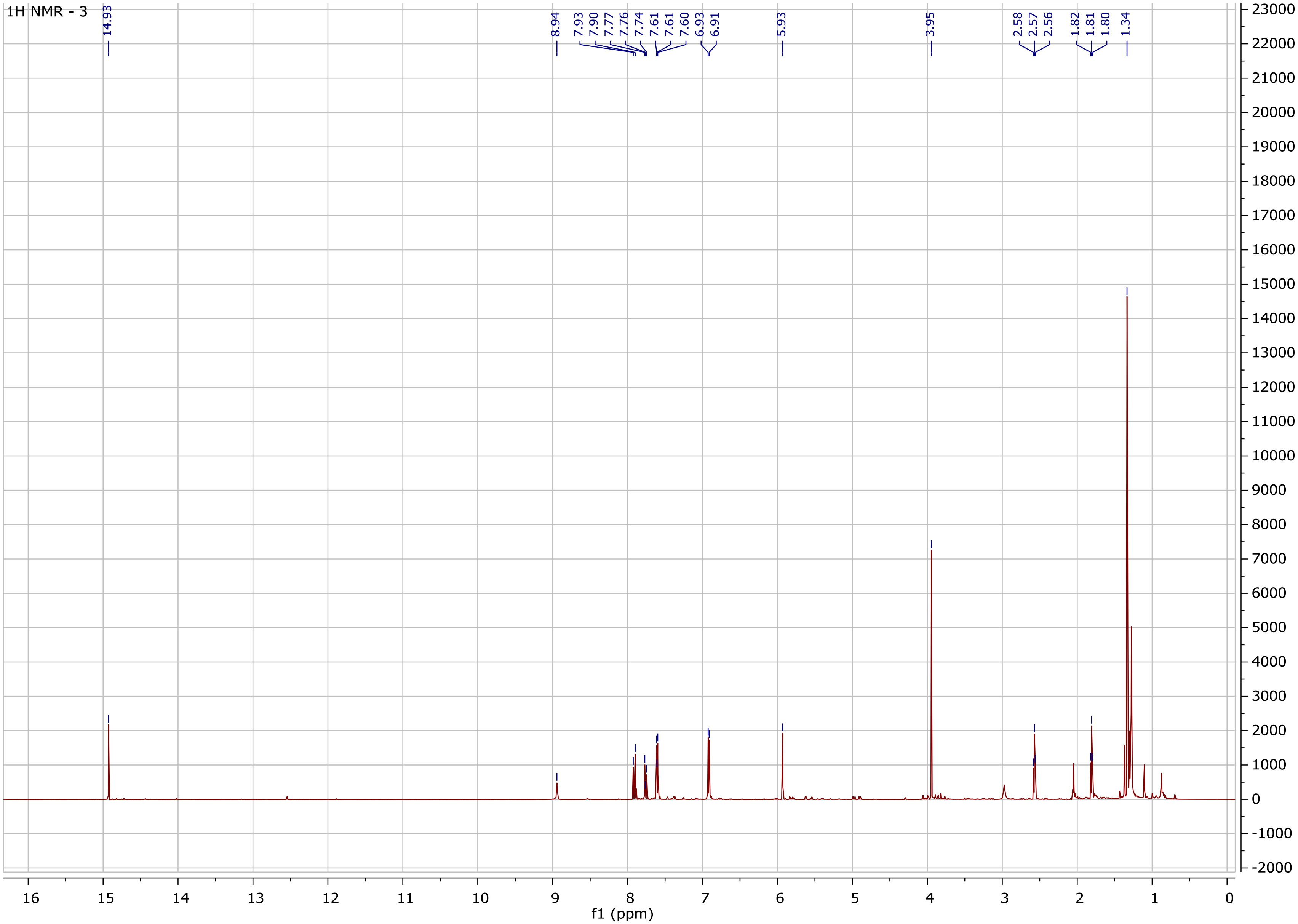
1H NMR - 3
14.93
8.94
7.93
7.90
7.77
7.76
7.74
7.61
7.61
7.60
6.93
6.91
5.93
3.95
2.58
2.57
2.56
1.82
1.81
1.80
1.34
f1 (ppm)

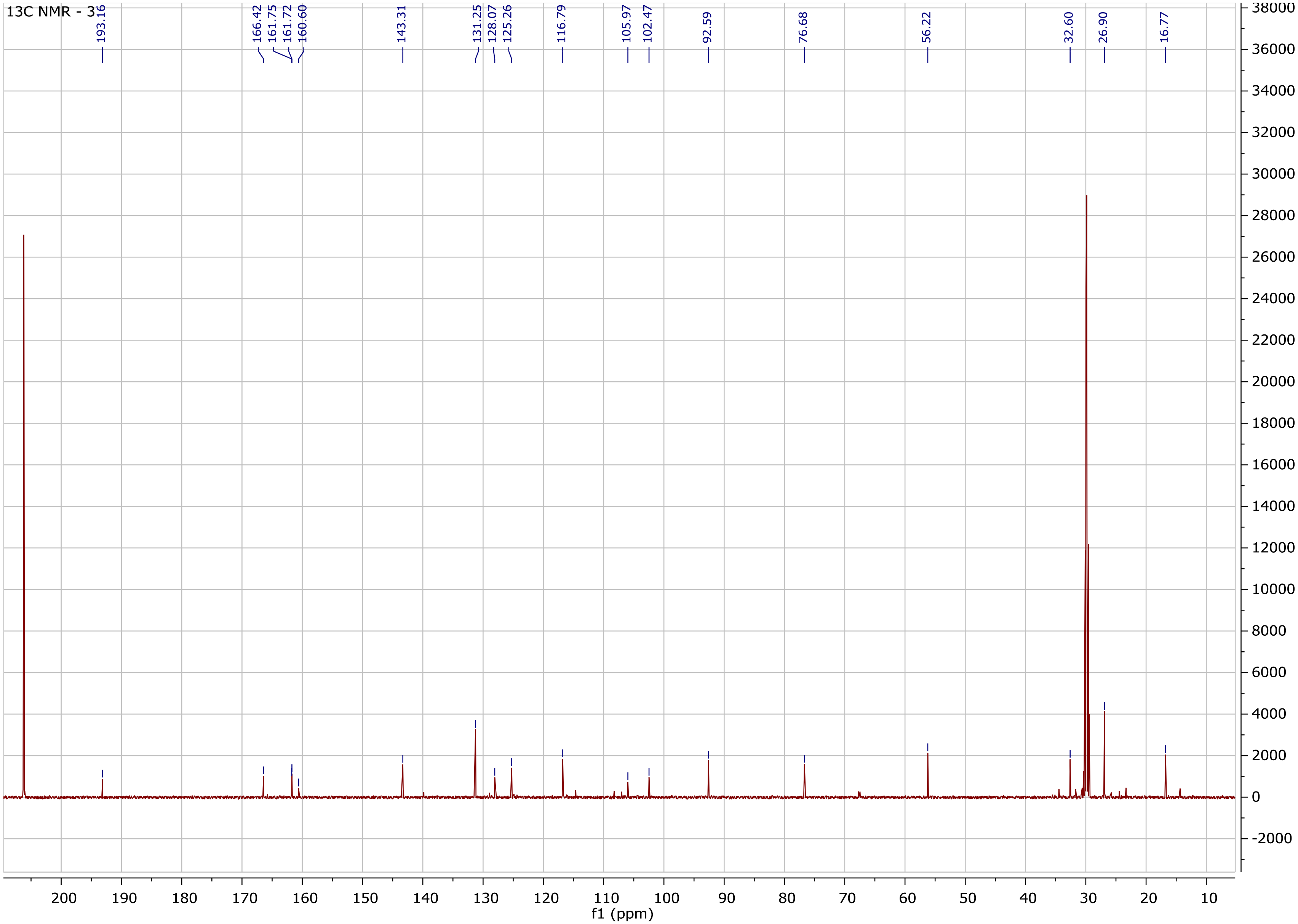
13C NMR - 3
193.16
166.42
161.75
161.72
160.60
143.31
131.25
128.07
125.26
116.79
105.97
102.47
92.59
76.68
56.22
32.60
26.90
16.77
f1 (ppm)

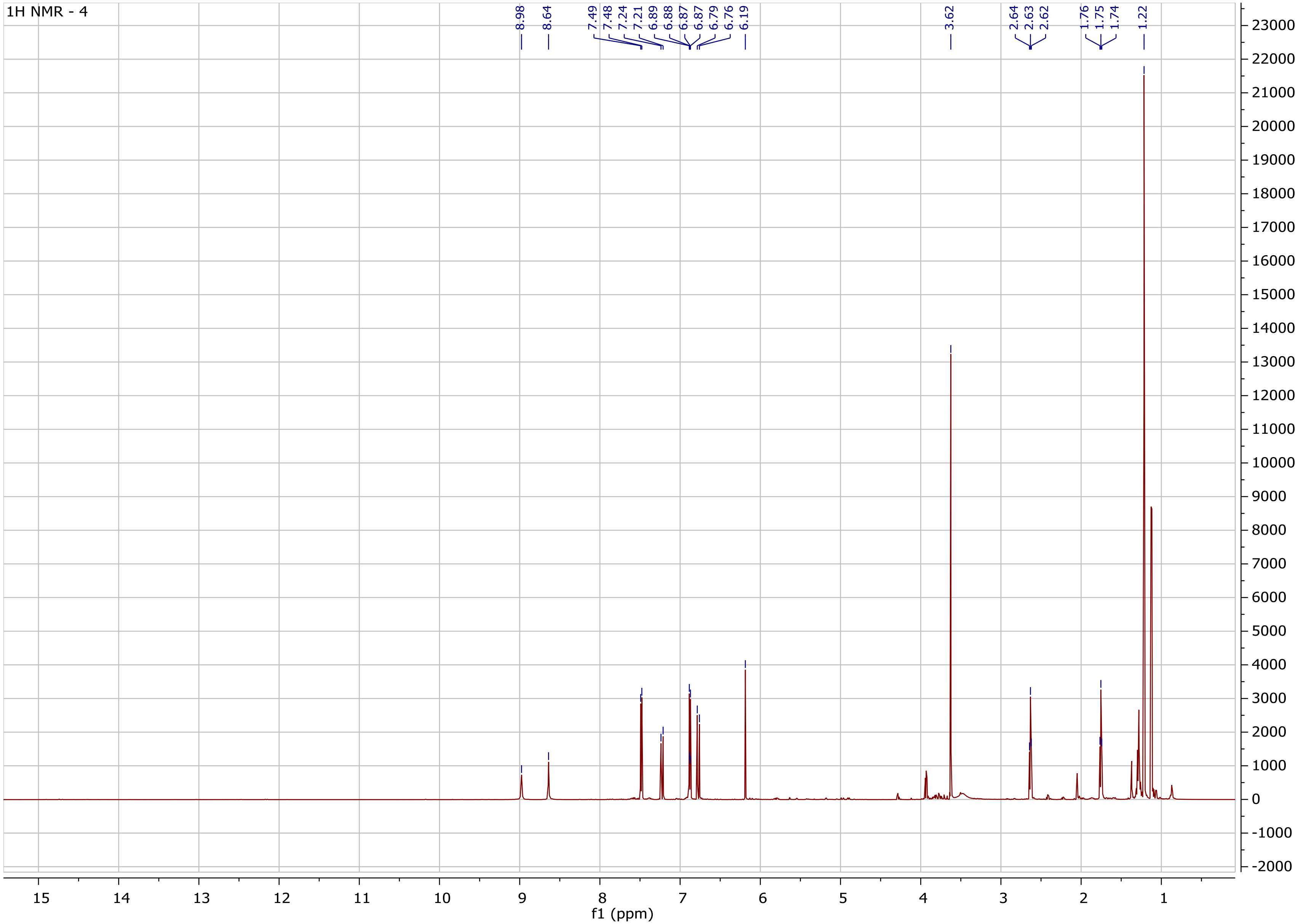
1H NMR - 4
8.98
8.64
7.49
7.48
7.24
7.21
6.89
6.88
6.87
6.87
6.79
6.76
6.19
3.62
2.64
2.63
2.62
1.76
1.75
1.74
1.22
f1 (ppm)

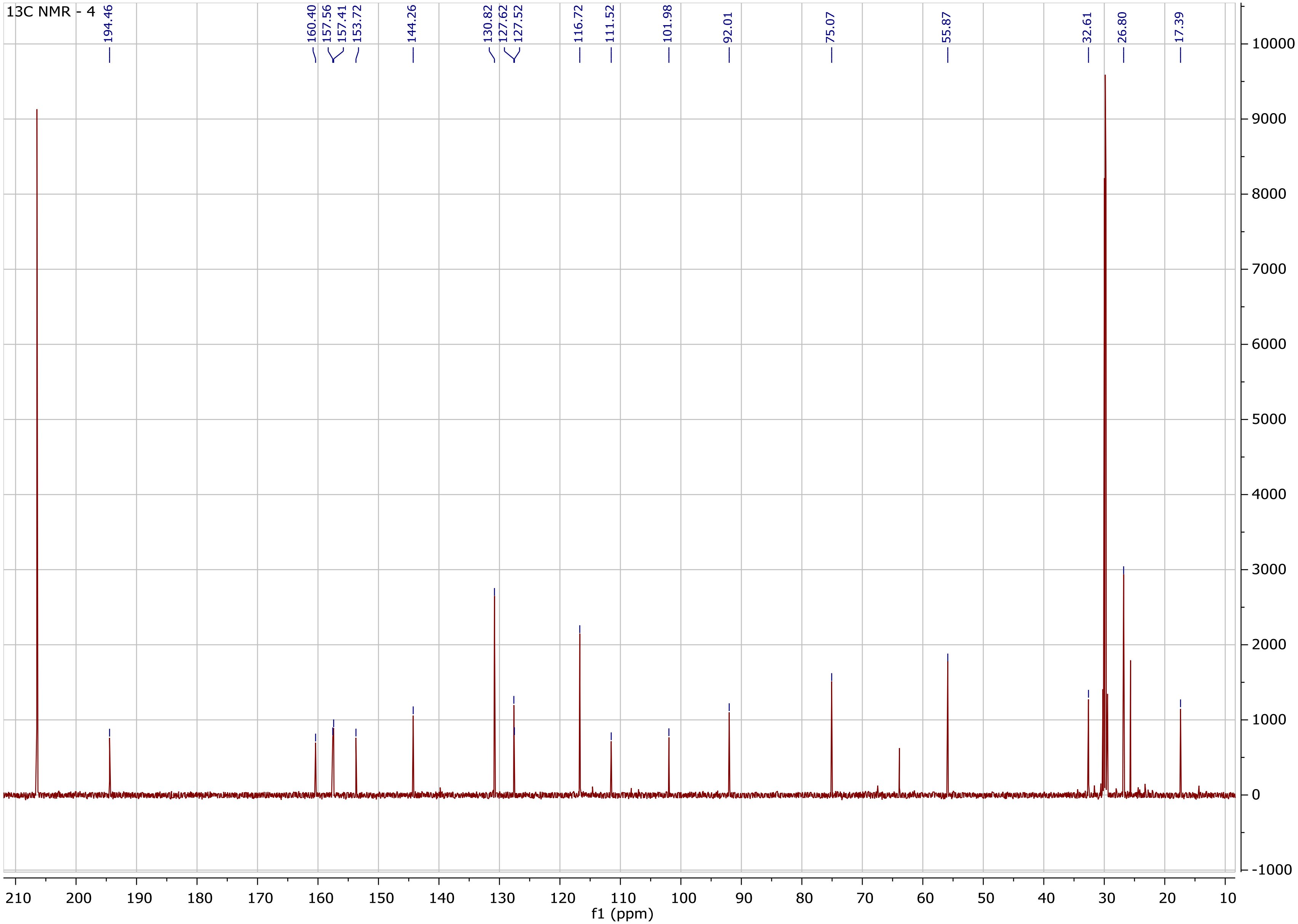
13C NMR - 4
194.46
160.40
157.56
157.41
153.72
144.26
130.82
127.62
127.52
116.72
111.52
101.98
92.01
75.07
55.87
32.61
26.80
17.39
f1 (ppm)

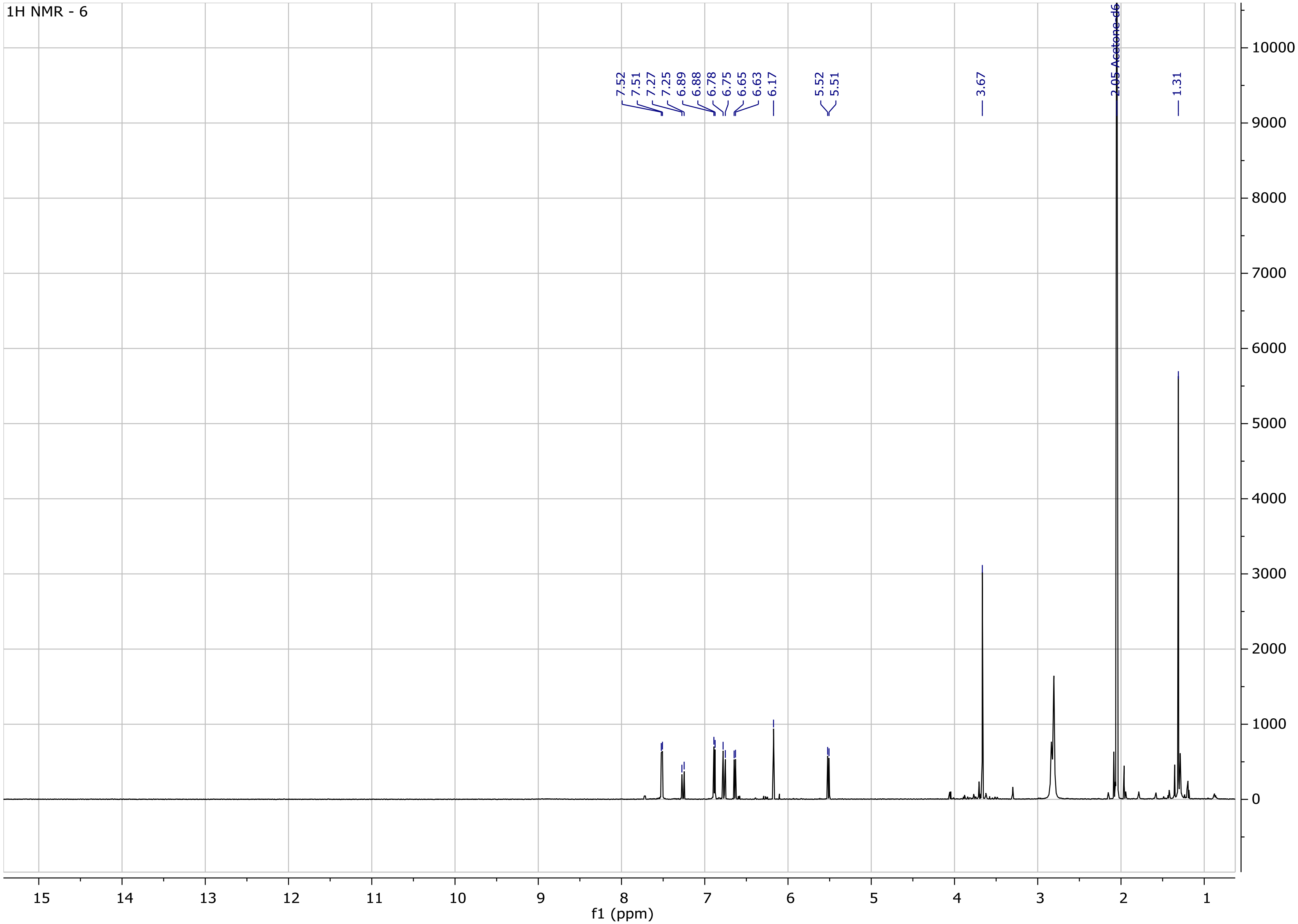
1H NMR - 6
7.52
7.51
7.27
7.25
6.89
6.88
6.78
6.75
6.65
6.63
6.17
5.52
5.51
3.67
2.05 Acetone-d6
1.31
f1 (ppm)

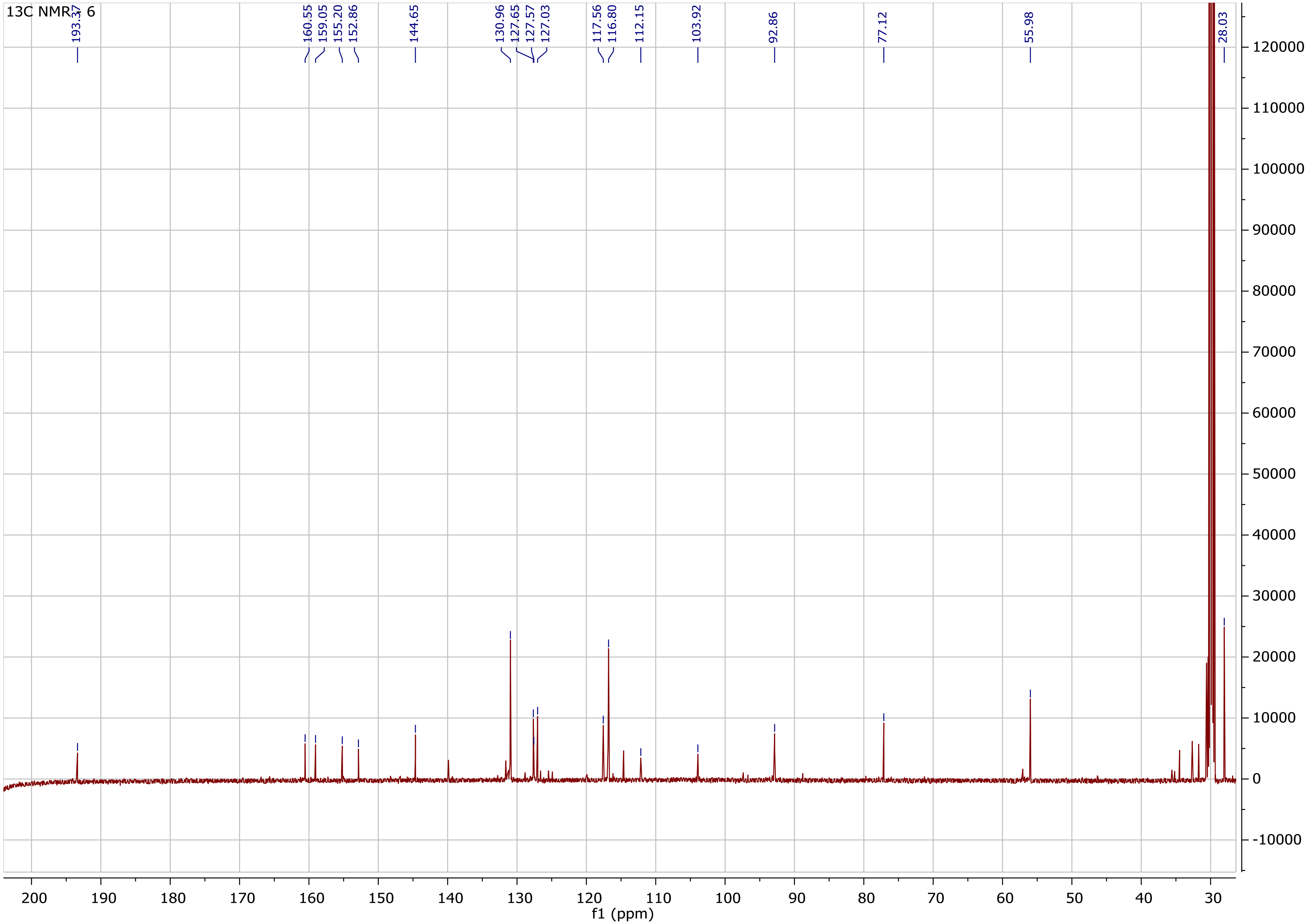
13C NMR- 6
193.37
160.55
159.05
155.20
152.86
144.65
130.96
127.65
127.57
127.03
117.56
116.80
112.15
103.92
92.86
77.12
55.98
28.03
f1 (ppm)

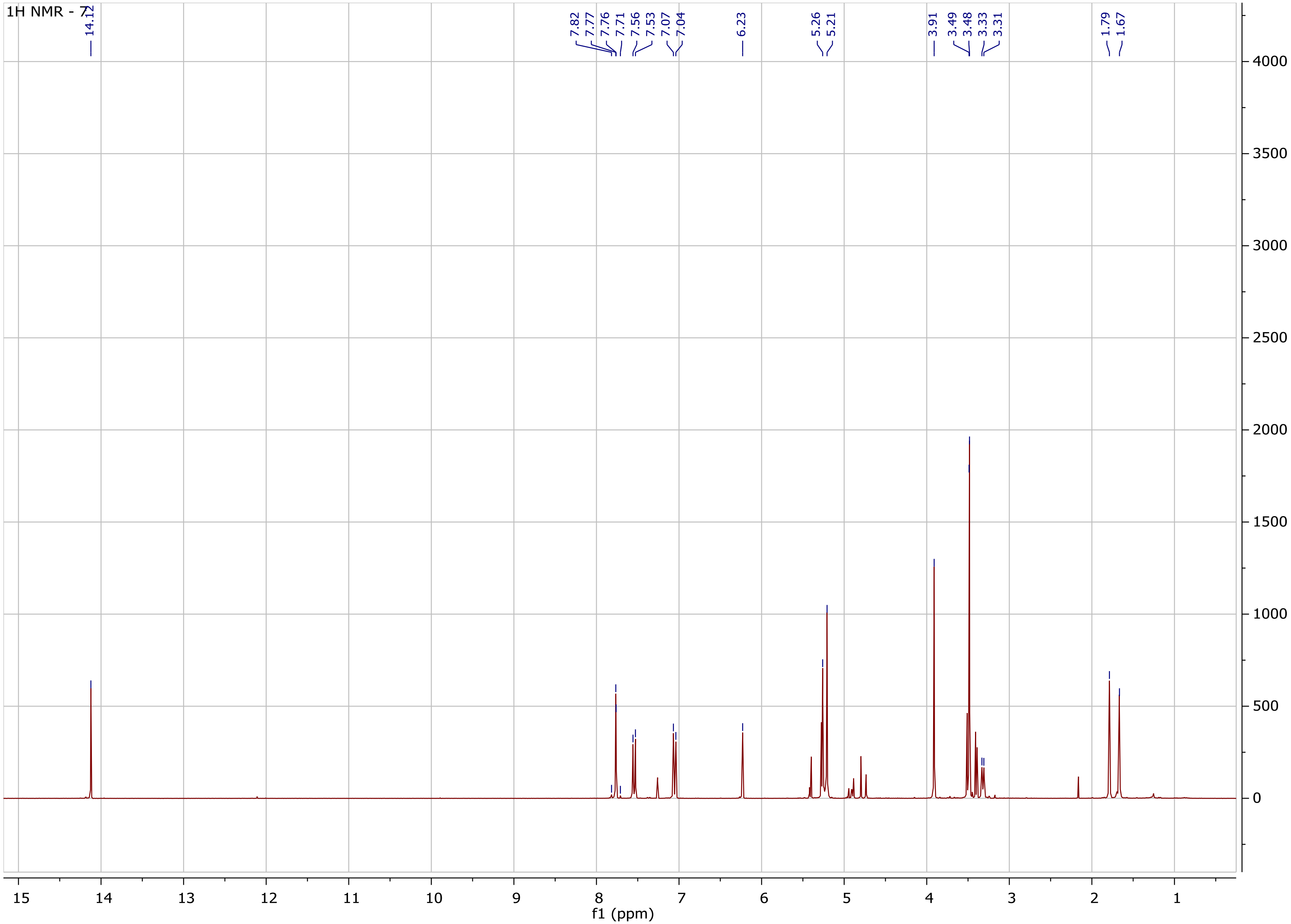
1H NMR - 7
14.12
7.82
7.77
7.76
7.71
7.56
7.53
7.07
7.04
6.23
5.26
5.21
3.91
3.49
3.48
3.33
3.31
1.79
1.67
f1 (ppm)

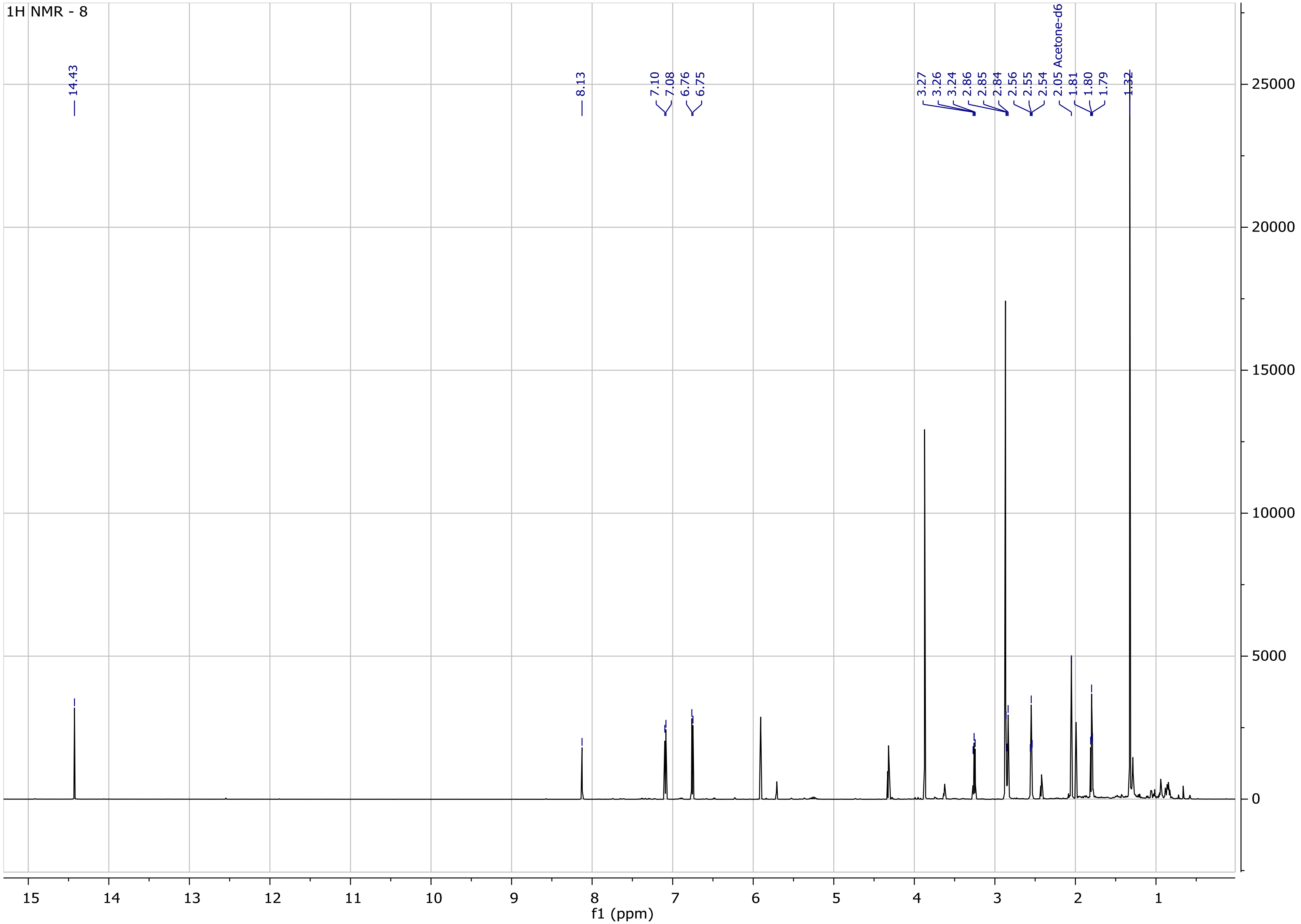
1H NMR - 8
14.43
8.13
7.10
7.08
6.76
6.75
3.27
3.26
3.24
2.86
2.85
2.84
2.56
2.55
2.54
2.05 Acetone-d6
1.81
1.80
1.79
1.32
25000
20000
15000
10000
5000
0
15 14 13 12 11 10 9 8 7 6 5 4 3 2 1
f1 (ppm)

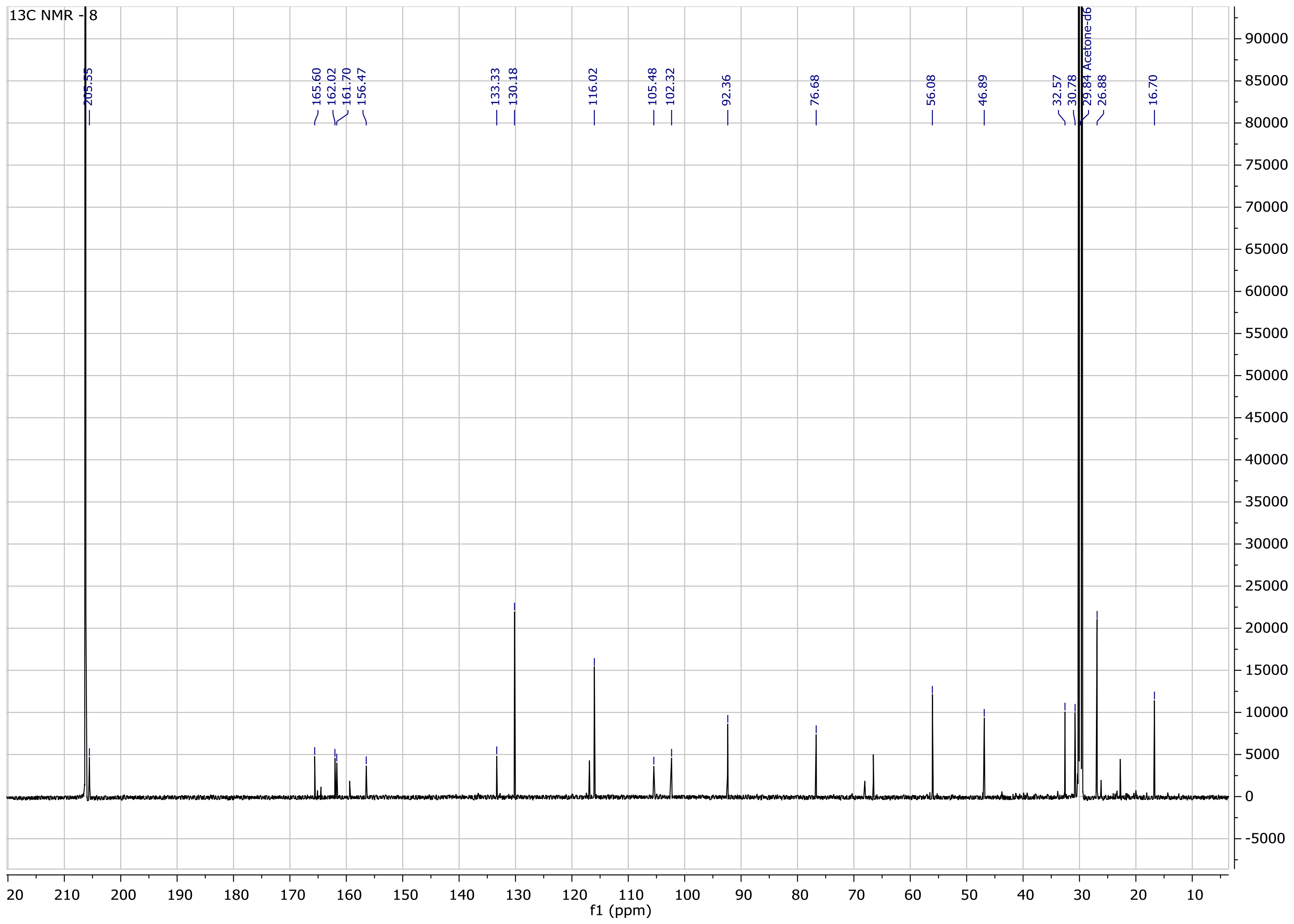
13C NMR - 8
205.55
165.60
162.02
161.70
156.47
133.33
130.18
116.02
105.48
102.32
92.36
76.68
56.08
46.89
32.57
30.78
29.84 Acetone-d6
26.88
16.70
f1 (ppm)

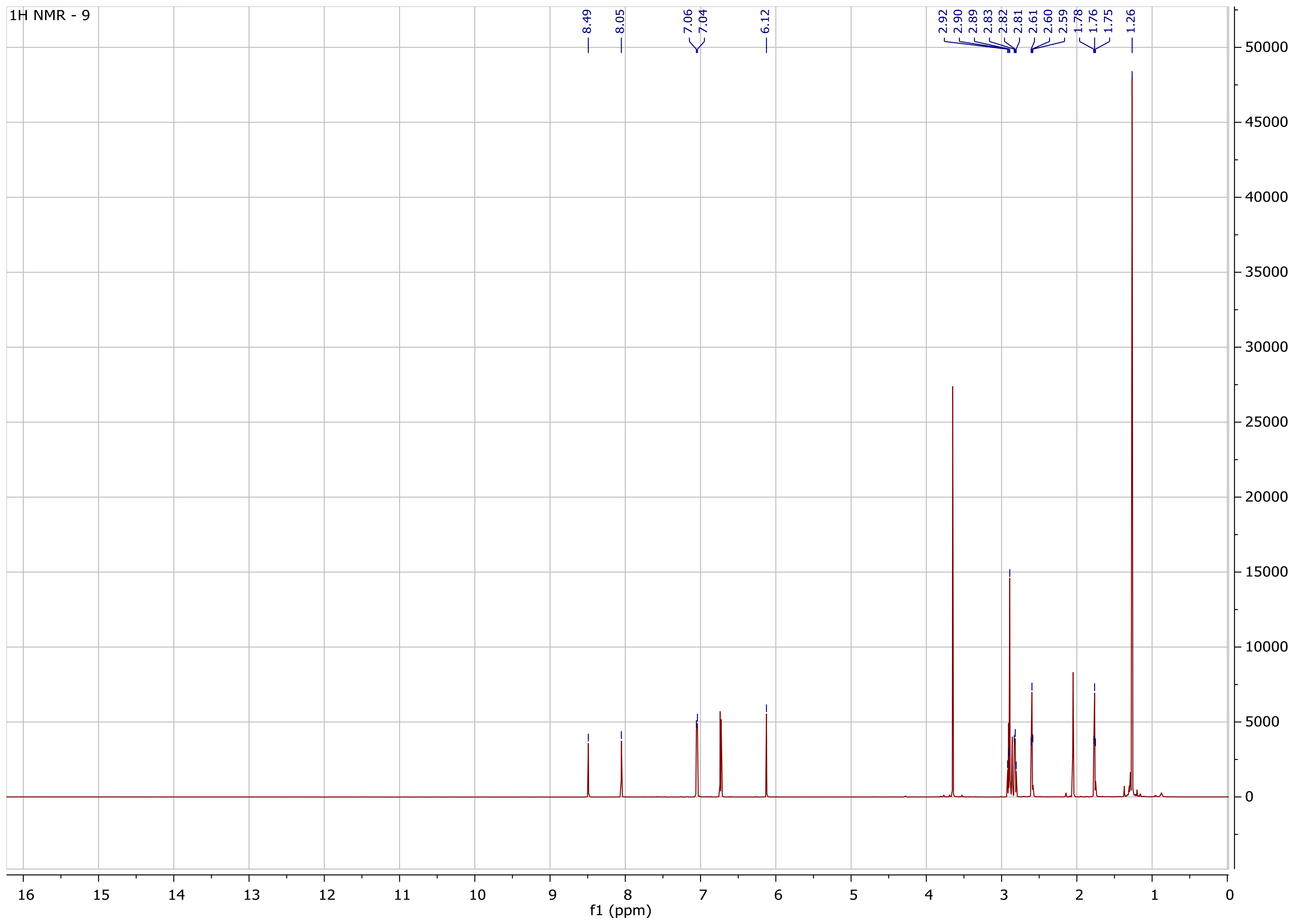
1H NMR - 9
8.49
8.05
7.06
7.04
6.12
2.92
2.90
2.89
2.83
2.82
2.81
2.61
2.60
2.59
1.78
1.76
1.75
1.26
f1 (ppm)

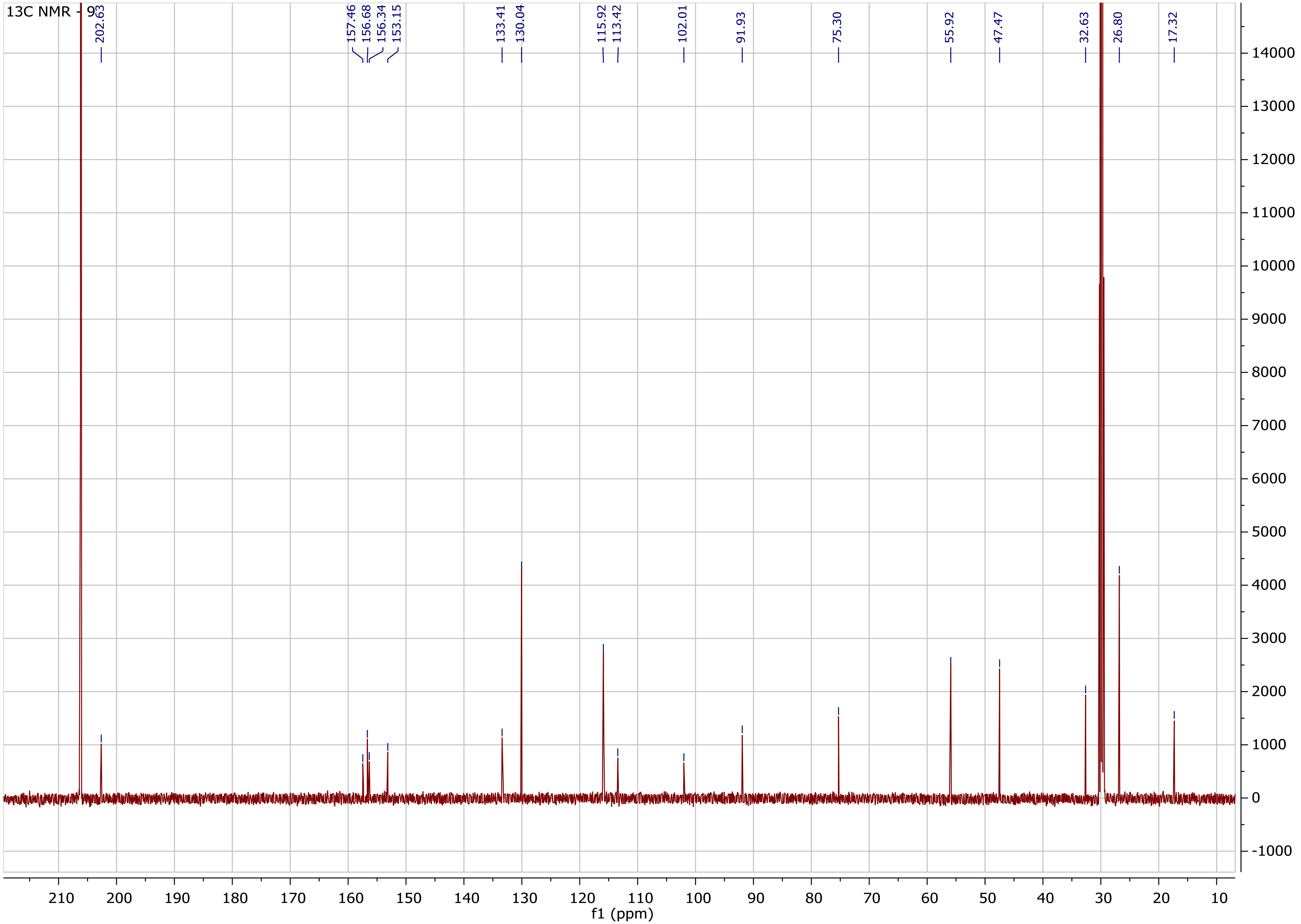
13C NMR - 9
202.63
157.46
156.68
156.34
153.15
133.41
130.04
115.92
113.42
102.01
91.93
75.30
55.92
47.47
32.63
26.80
17.32
f1 (ppm)

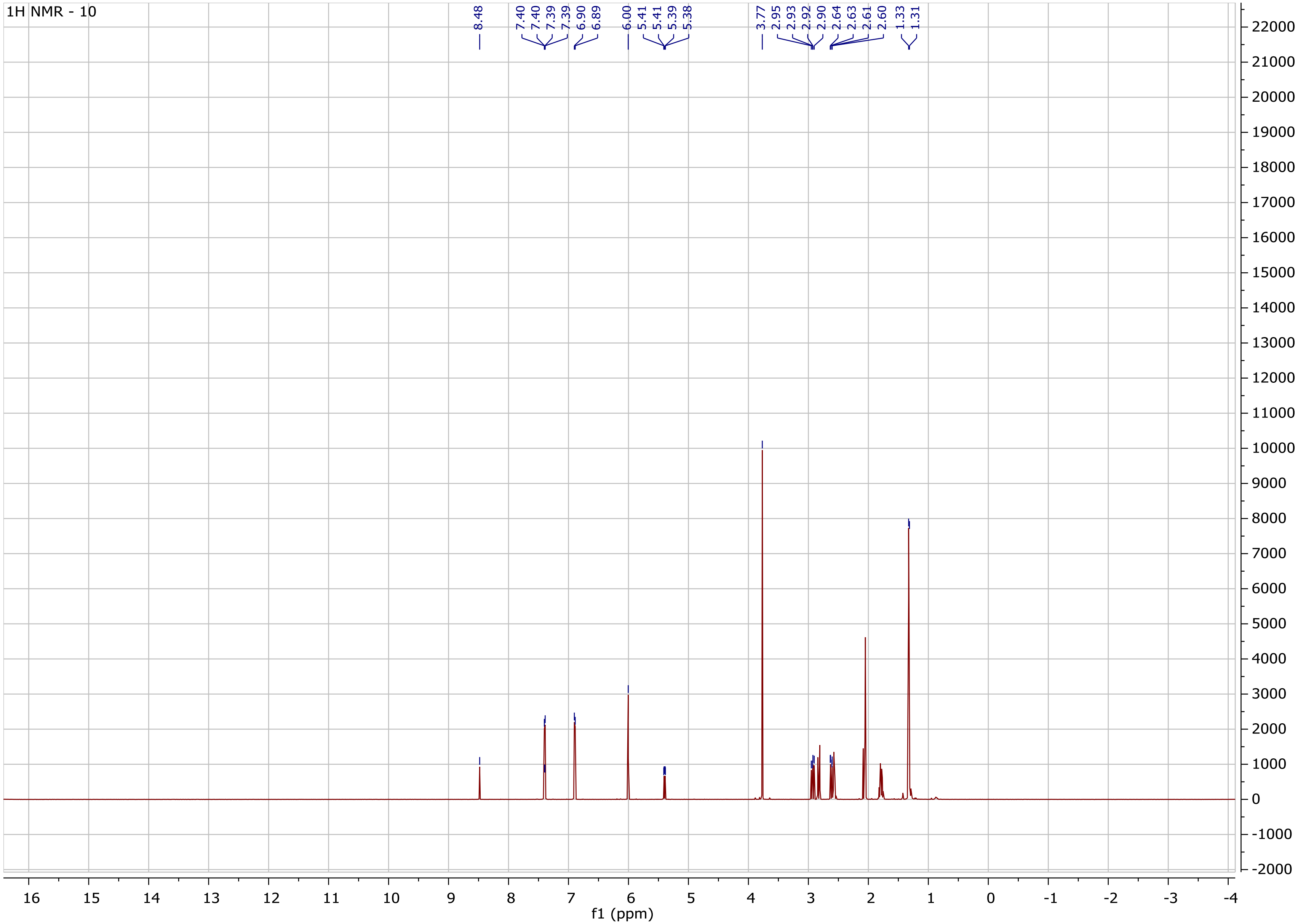

1H NMR - 10
8.48
7.40
7.40
7.39
7.39
6.90
6.89
6.00
5.41
5.41
5.39
5.38
3.77
2.95
2.93
2.92
2.90
2.64
2.63
2.61
2.60
1.33
1.31
f1 (ppm)

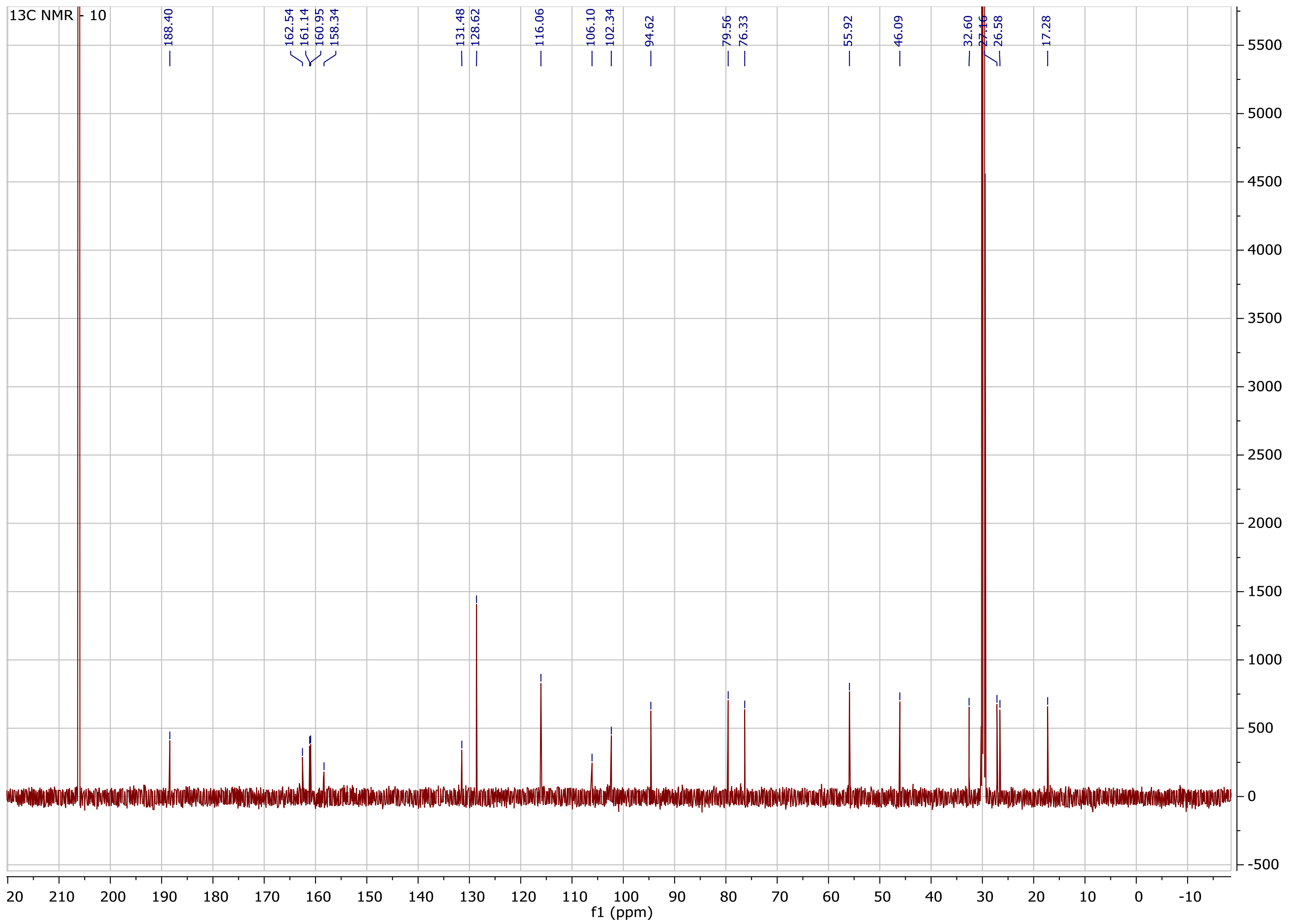
13C NMR - 10
188.40
162.54
161.14
160.95
158.34
131.48
128.62
116.06
106.10
102.34
94.62
79.56
76.33
55.92
46.09
32.60
27.16
26.58
17.28
f1 (ppm)

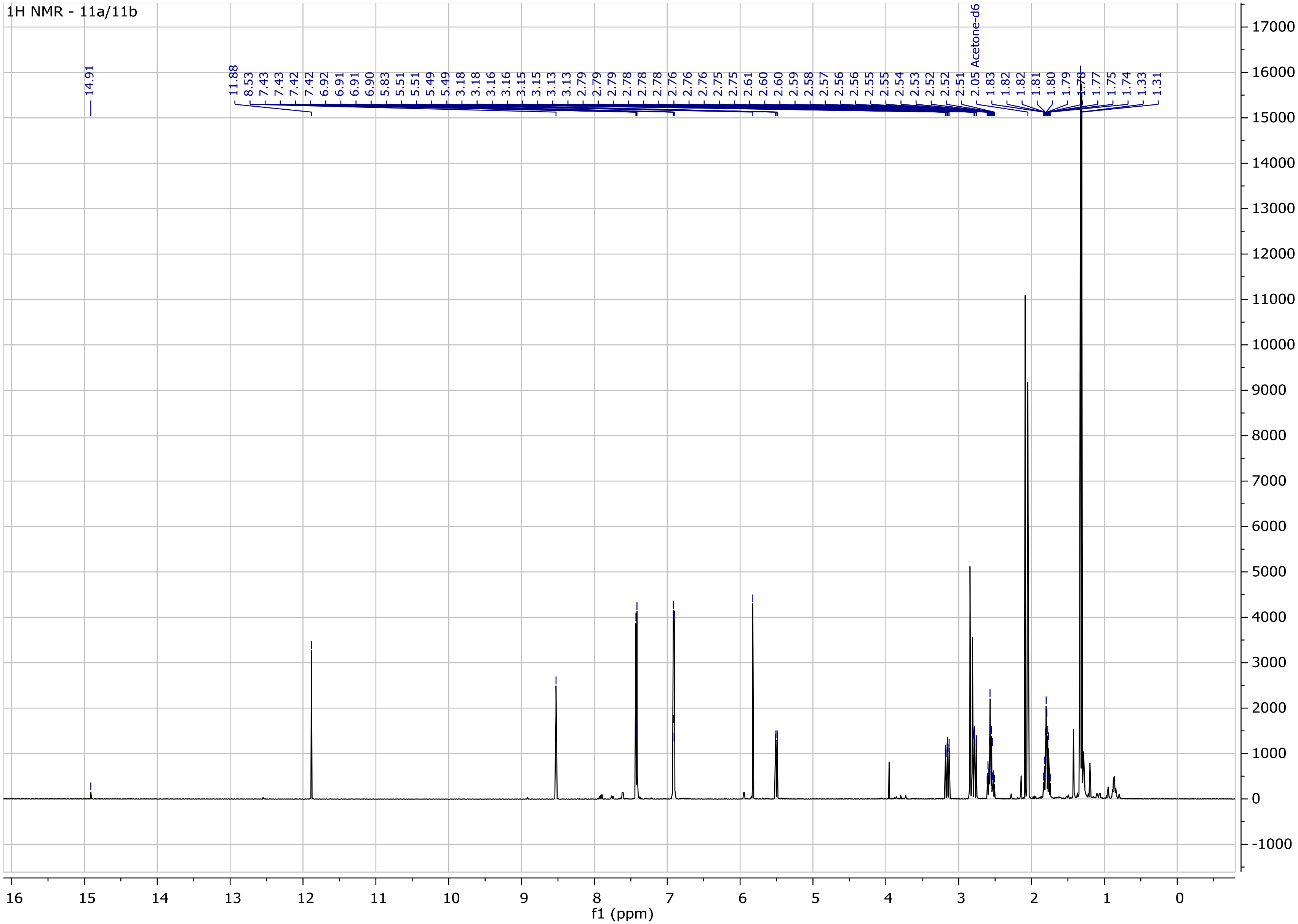
1H NMR - 11a/11b
14.91
11.88 8.53 7.43 7.43 7.42 7.42 6.92 6.91 6.91 6.90 5.83 5.51 5.51 5.49 5.49 3.18 3.18 3.16 3.16 3.15 3.15 3.13 3.13 2.79 2.79 2.79 2.78 2.78 2.78 2.76 2.76 2.76 2.75 2.75 2.61 2.60 2.60 2.59 2.58 2.57 2.56 2.56 2.55 2.55 2.54 2.53 2.52 2.52 2.51 2.05 Acetone-d6 1.83 1.82 1.82 1.81 1.80 1.79 1.78 1.77 1.75 1.74 1.33 1.31
f1 (ppm)

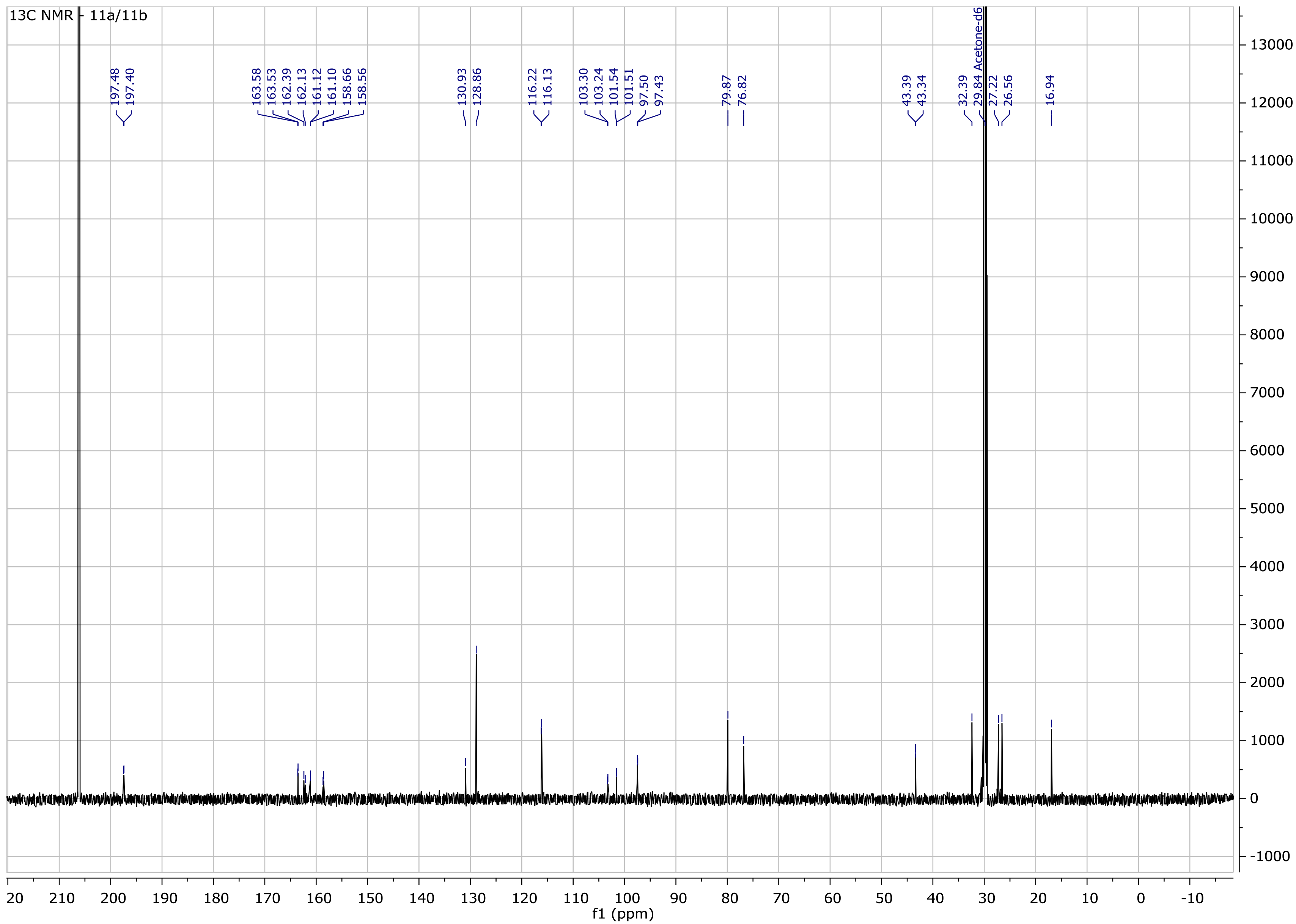

13C NMR - 11a/11b
197.48
197.40
163.58
163.53
162.39
162.13
161.12
161.10
158.66
158.56
130.93
128.86
116.22
116.13
103.30
103.24
101.54
101.51
97.50
97.43
79.87
76.82
43.39
43.34
32.39
29.84 Acetone-d6
27.22
26.56
16.94
f1 (ppm)

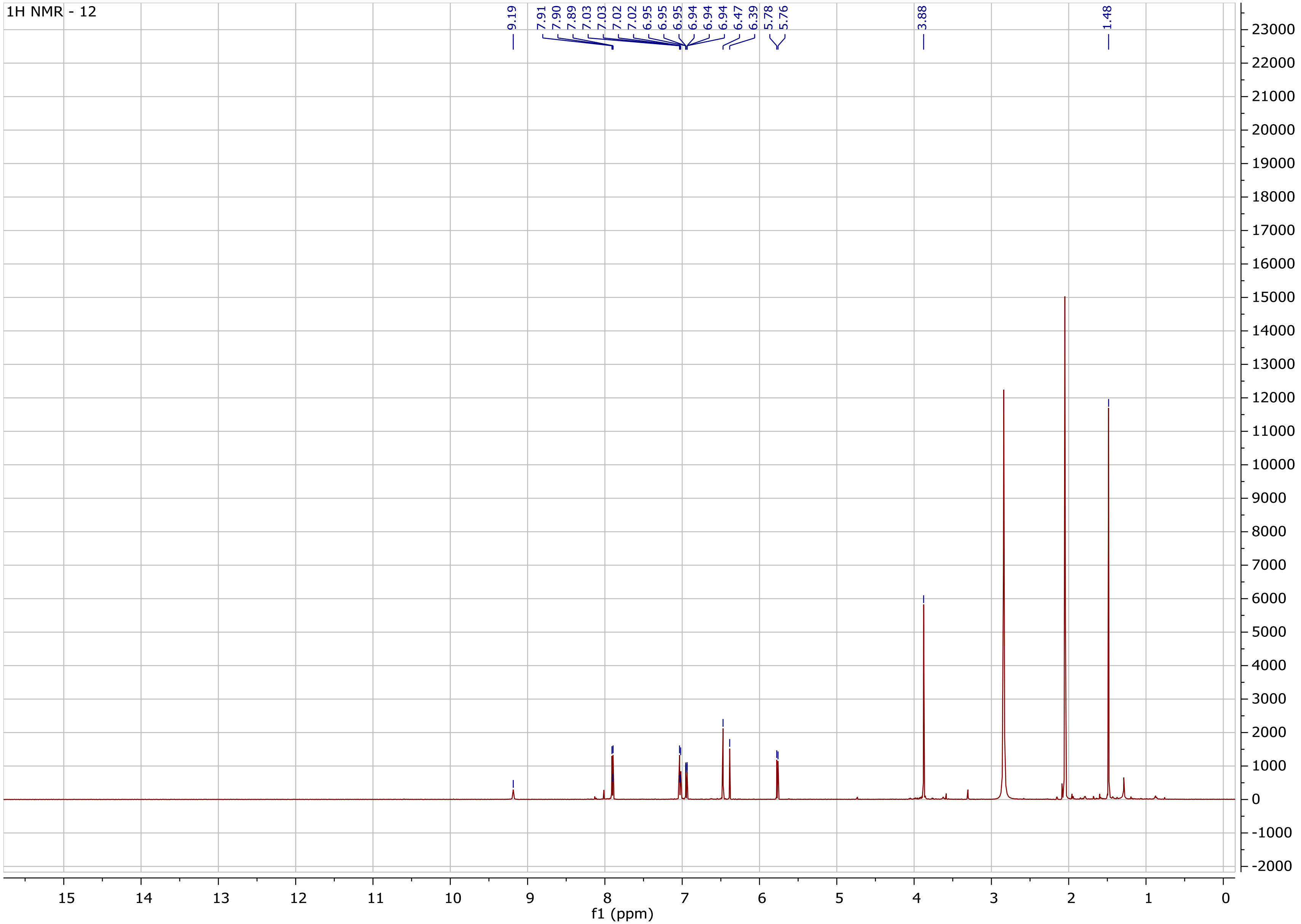
1H NMR - 12
9.19
7.91
7.90
7.89
7.03
7.02
7.02
6.95
6.95
6.94
6.94
6.94
6.47
6.39
5.78
5.76
3.88
1.48
f1 (ppm)

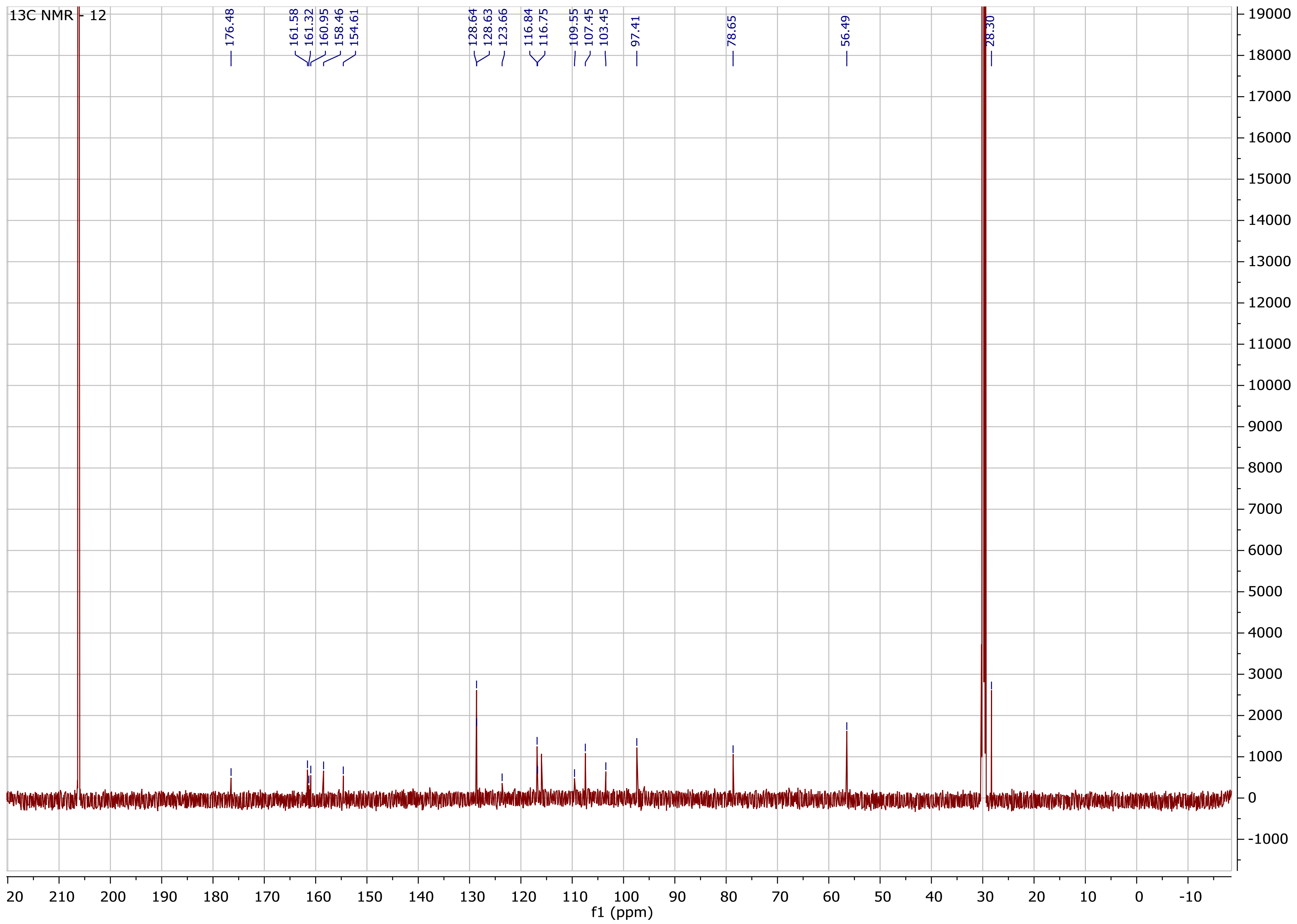

13C NMR - 12
176.48
161.58
161.32
160.95
158.46
154.61
128.64
128.63
123.66
116.84
116.75
109.55
107.45
103.45
97.41
78.65
56.49
28.30
f1 (ppm)

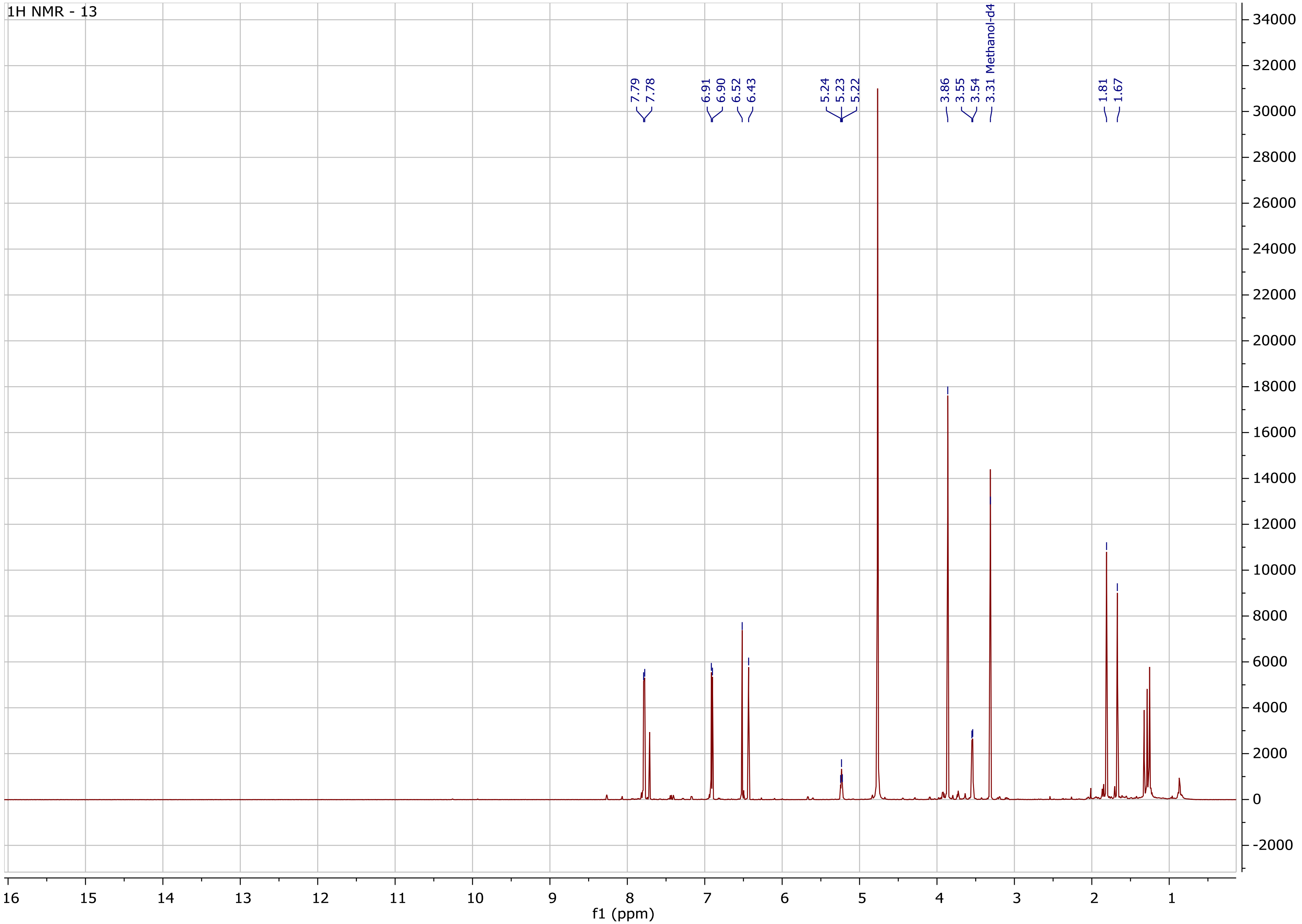
1H NMR - 13
7.79
7.78
6.91
6.90
6.52
6.43
5.24
5.23
5.22
3.86
3.55
3.54
3.31 Methanol-d4
1.81
1.67
f1 (ppm)

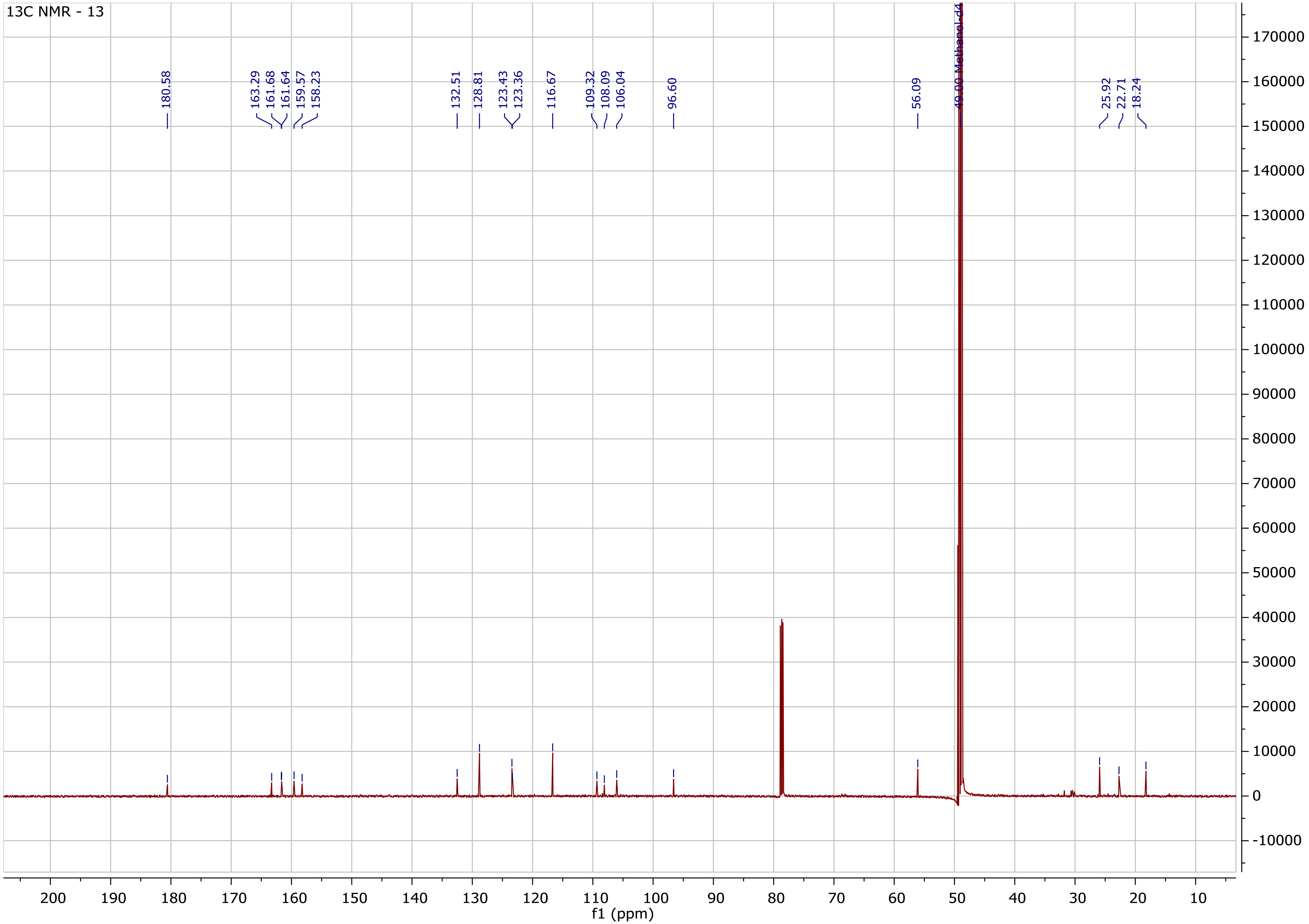

13C NMR - 13
180.58
163.29
161.68
161.64
159.57
158.23
132.51
128.81
123.43
123.36
116.67
109.32
108.09
106.04
96.60
56.09
49.00 Methanol-d4
25.92
22.71
18.24
f1 (ppm)

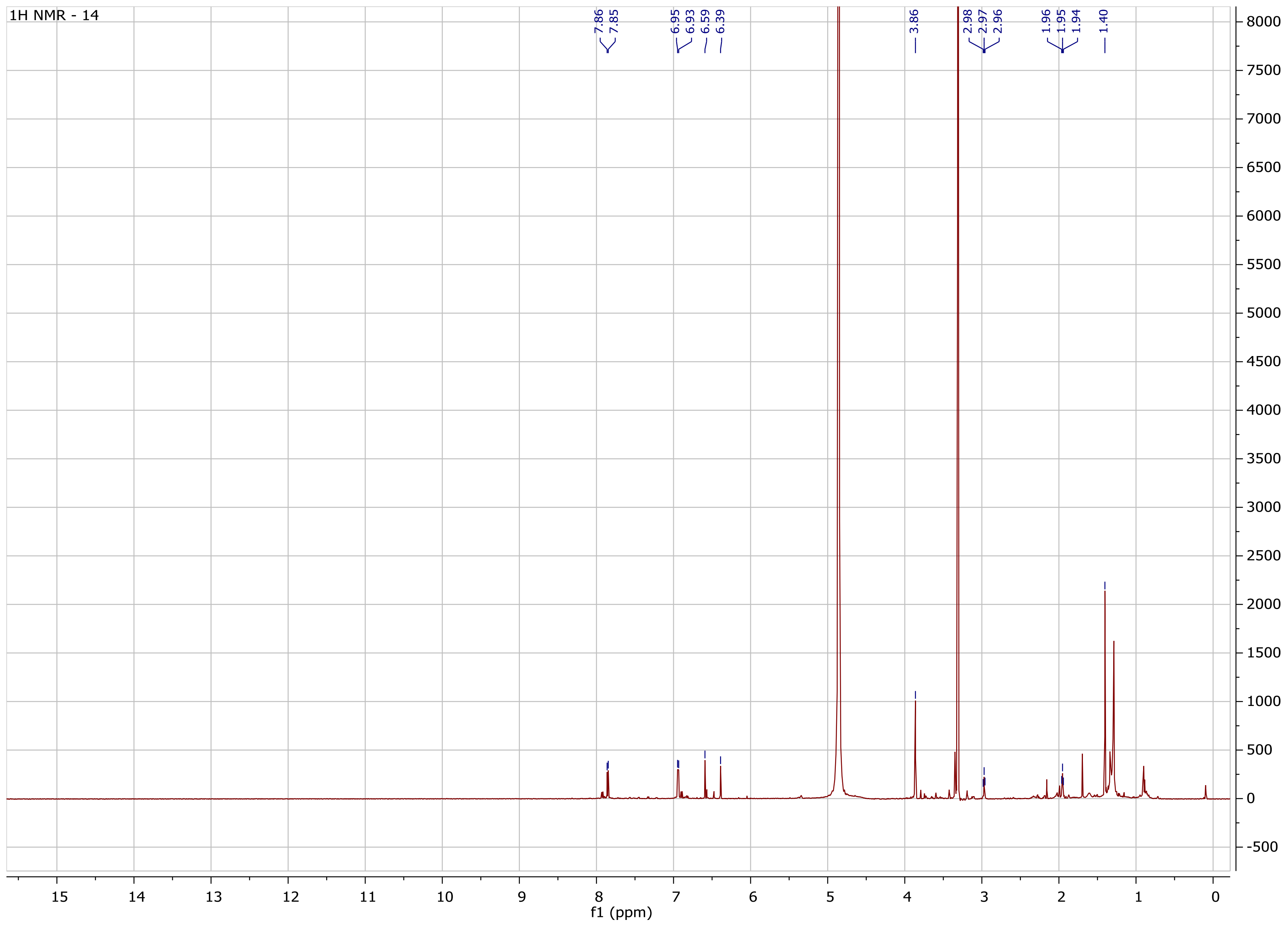
1H NMR - 14
7.86
7.85
6.95
6.93
6.59
6.39
3.86
2.98
2.97
2.96
1.96
1.95
1.94
1.40
f1 (ppm)

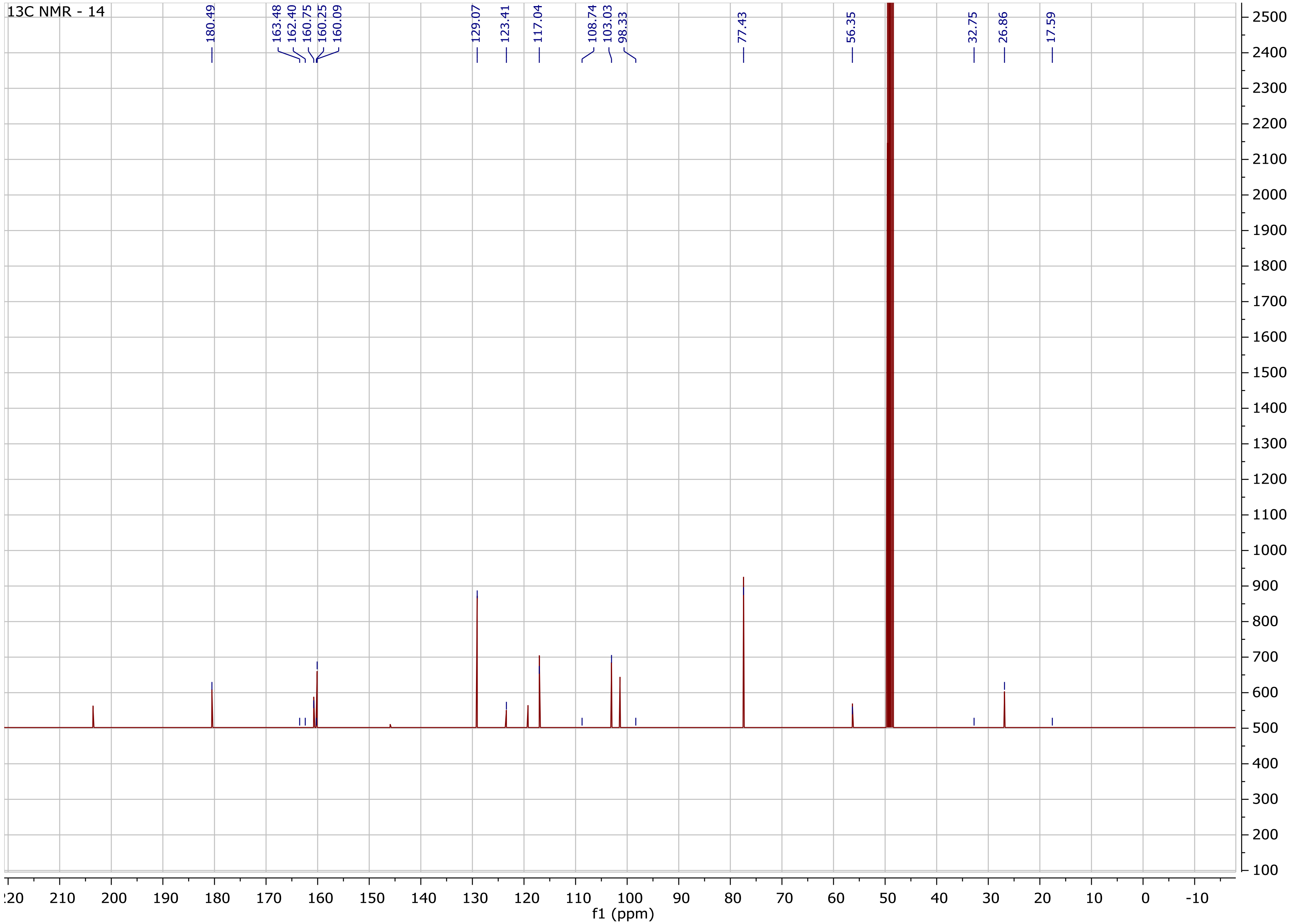

13C NMR - 14
180.49
163.48
162.40
160.75
160.25
160.09
129.07
123.41
117.04
108.74
103.03
98.33
77.43
56.35
32.75
26.86
17.59
f1 (ppm)
